# Transcriptional control of morphological properties of direction-selective T4/T5 neurons in *Drosophila*

**DOI:** 10.1242/dev.169763

**Published:** 2019-01-29

**Authors:** Tabea Schilling, Aicha H. Ali, Aljoscha Leonhardt, Alexander Borst, Jesús Pujol-Martí

**Affiliations:** Department of ‘Circuits – Computation – Models’, Max Planck Institute of Neurobiology, 82152 Martinsried, Germany

**Keywords:** Neural development, Layer specificity, Optic lobe, *Drosophila*, SoxN, Sox102F

## Abstract

In the *Drosophila* visual system, T4/T5 neurons represent the first stage of computation of the direction of visual motion. T4 and T5 neurons exist in four subtypes, each responding to motion in one of the four cardinal directions and projecting axons into one of the four lobula plate layers. However, all T4/T5 neurons share properties essential for sensing motion. How T4/T5 neurons acquire their properties during development is poorly understood. We reveal that the transcription factors SoxN and Sox102F control the acquisition of properties common to all T4/T5 neuron subtypes, i.e. the layer specificity of dendrites and axons. Accordingly, adult flies are motion blind after disruption of *SoxN* or *Sox102F* in maturing T4/T5 neurons. We further find that the transcription factors Ato and Dac are redundantly required in T4/T5 neuron progenitors for SoxN and Sox102F expression in T4/T5 neurons, linking the transcriptional programmes specifying progenitor identity to those regulating the acquisition of morphological properties in neurons. Our work will help to link structure, function and development in a neuronal type performing a computation that is conserved across vertebrate and invertebrate visual systems.

## Abstract (precis)

**Summary:** RNA interference identifies SoxN and Sox102F as key regulators of the morphological properties of a neuronal type of the fruit fly visual system that computes the direction of motion.

## INTRODUCTION

**Fig. 1.**
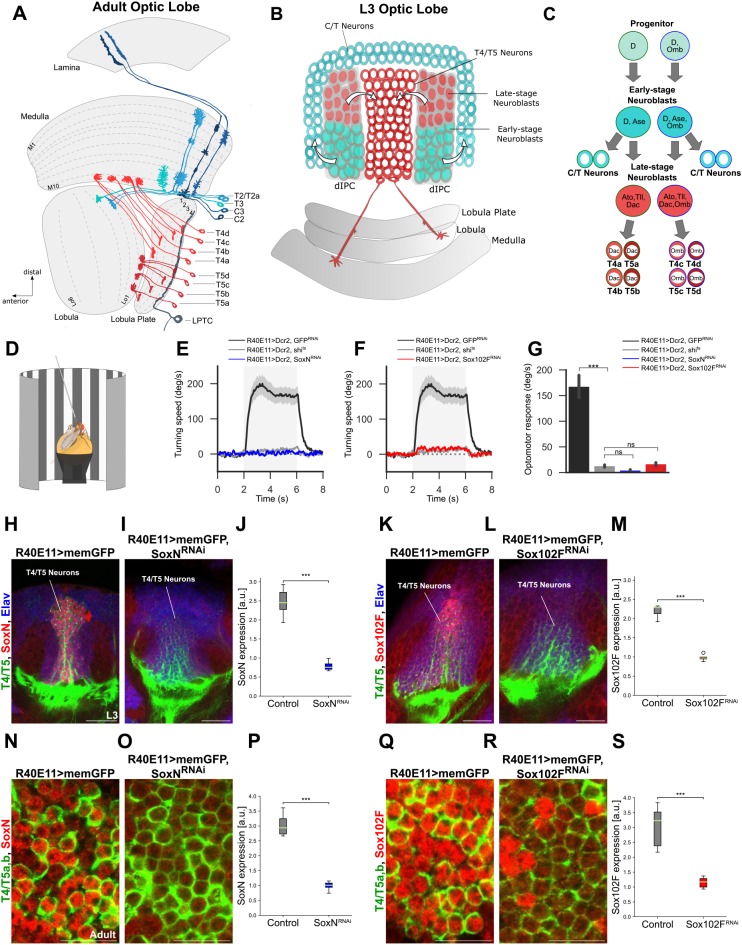
***SoxN* or *Sox102F* knockdown in T4/T5 neurons impairs the optomotor response.** (A) Schematic of adult optic lobe (dorsal view) highlighting T4/T5 neuron subtypes (a,b,c,d). C/T neurons (T2, T2a, T3, C2 and C3) and a lobula plate tangential cell (LPTC) with dendrites in lobula plate layer 4 receiving input from T4/T5_d_ axons are also shown. (B) Schematic of L3 larval optic lobe highlighting early- and late-stage dIPC neuroblasts, and their offspring C/T and T4/T5 neurons. (C) Summary of transcription factors expressed in early- and late-stage dIPC neuroblasts. (D) Set-up used for measuring the optomotor response of adult flies. (E,F) Average turning speeds in response to rotation of a grating pattern (grey shaded areas) of flies expressing *GFP-RNAi* (negative control), *shi^ts^* (positive control, T4/T5 block), *SoxN-RNAi* or *Sox102F-RNAi* in T4/T5 neurons (*n*=10 flies per group). (G) Average optomotor responses of flies expressing *GFP-RNAi*, *shi^ts^*, *SoxN-RNAi* or *Sox102F-RNAi* in T4/T5 neurons (*n*=10 flies per group). (H-S) SoxN and Sox102F expression in late L3 larval and adult optic lobes with wild-type T4/T5 neurons, and with T4/T5 neurons expressing *SoxN-RNAi* or *Sox102F-RNAi.* T4/T5 neurons were labelled with membrane-targeted GFP (memGFP). Neuronal somata in H-L were marked with anti-Elav. Quantifications of SoxN and Sox102F levels in T4/T5 somata are shown in J,M,P,S (*n*=4-11 optic lobes per group; a.u., arbitrary units). ns, not significant (*P*>0.05); ***P<0.001. Scale bars: 20 µm (H,I,K,L); 10 µm (N,O,Q,R).

The formation of neural circuits comprising neurons with specific morphological and physiological properties is key for the proper function of the brain. The *Drosophila* optic lobe has emerged as a powerful model in which to study this process. It consists of four neuropils downstream of the retina: lamina, medulla, lobula and lobula plate, all made of repeating columns that process signals from specific points in space and are arranged in a retinotopic fashion. In addition, the medulla, lobula and lobula plate are subdivided into layers that process distinct visual features in parallel (Maisak et al., 2013; Strother et al., 2014). The four neuropils of the optic lobe contain more than 100 different neuronal types (Fischbach and Dittrich, 1989), some of which have been studied in great anatomical and functional detail. Prominent examples are T4 and T5 neurons, the local motion detectors in *Drosophila* (Maisak et al., 2013). Whereas T4 neurons have their dendrites in the medulla and receive input from neurons encoding brightness increments, T5 dendrites arborise in the lobula and receive input from neurons encoding brightness decrements (Joesch et al., 2010; Maisak et al., 2013; Shinomiya et al., 2014; Takemura et al., 2017). Apart from this difference, T4 and T5 neurons share many morphological and functional properties (Shinomiya et al., 2015). Remarkably, their dendrites extend across a similar number of columns, are confined to a specific layer of their target neuropil (Fig. 1A) (Fischbach and Dittrich, 1989), and use a common mechanism to compute local motion from the signals of columnar, non-direction-selective neurons (Haag et al., 2016, 2017). Interestingly, T4 and T5 neurons exist in four subtypes (a, b, c and d), each responding exclusively to motion in one of the four cardinal directions (front-to-back, back-to-front, upwards and downwards) (Maisak et al., 2013). Axons from T4 and T5 neurons of the same subtype terminate specifically in one of four lobula plate layers (Fig. 1A) (Fischbach and Dittrich, 1989; Maisak et al., 2013). There, they establish synapses with the dendrites of wide-field, direction-selective lobula plate tangential cells (Joesch et al., 2008; Mauss et al., 2014; Schnell et al., 2010), some of which are also restricted to a single lobula plate layer (Boergens et al., 2018; Scott et al., 2002). How T4/T5 neurons acquire these properties during development to establish a map of directional tuning is poorly understood.

T4/T5 neurons originate from a progenitor domain in the developing fly brain known as the inner proliferation centre (IPC) (Hofbauer and Campos-Ortega, 1990; Oliva et al., 2014). In a process that extends from late second instar larval stage to early pupal stage, neuroepithelial cells from the proximal IPC (pIPC) progressively become progenitors that migrate to a second zone, the distal IPC (dIPC), where they assume a neuroprogenitor (neuroblast in *Drosophila*) fate (Apitz and Salecker, 2015; Hofbauer and Campos-Ortega, 1990; Nassif et al., 2003; Ngo et al., 2017). Neuroblasts in the dIPC transit through two temporal stages. Early-stage dIPC neuroblasts express Dichaete (D) and Asense (Ase), and generate ganglion mother cells that eventually produce postmitotic C2, C3, T2, T2a and T3 neurons (also known as C/T neurons) (Apitz and Salecker, 2015). Late-stage dIPC neuroblasts express Tailless (Tll), Atonal (Ato) and Dachshund (Dac), and produce ganglion mother cells that are the precursors of postmitotic T4/T5 neurons (Fig. 1B,C) (Apitz and Salecker, 2015; Mora et al., 2018; Oliva et al., 2014; Pinto-Teixeira et al., 2018). Therefore, temporal patterning of dIPC neuroblasts contributes to the specification of C/T versus T4/T5 neuron fate. Two recent studies have uncovered the mechanisms specifying T4 versus T5 identity and the identity of the four T4/T5 neuron subtypes (Apitz and Salecker, 2018; Pinto-Teixeira et al., 2018). These mechanisms involve spatial patterning in the pIPC neuroepithelium and Notch-dependent binary fate choices during the divisions of the neuroblast and ganglion mother cell precursors of T4/T5 neurons. In contrast to our current understanding regarding the specification of T4/T5 neuron progenitor identity, very little is known about how this translates into the acquisition of structural and functional properties in postmitotic T4/T5 neurons.

Each newborn T4 and T5 neuron must initiate gene expression programmes to terminally differentiate, i.e. to express a unique combination of effector genes defining its identity and function (Hobert, 2011). How are these effector genes selected during development? One possibility is that transcription factors expressed in T4/T5 neuron progenitors are inherited through successive cell divisions to regulate the acquisition of terminal characters in postmitotic T4/T5 neurons. This is the case for Optomotor-blind (Omb; also known as Bifid), which is expressed in dIPC neuroblasts and ganglion mother cells producing T4/T5_c,d_ neurons as a result of pIPC neuroepithelium spatial patterning. Omb is further maintained in maturing T4/T5_c,d_ neurons to endow them with subtype-specific terminal characters (Fig. 1C) (Apitz and Salecker, 2018). As a complementary mechanism, transcription factors transiently expressed in T4/T5 neuron progenitors might start a transcriptional cascade to control the acquisition of terminal properties in postmitotic T4/T5 neurons. Ato is transiently expressed in late-stage dIPC neuroblasts (Apitz and Salecker, 2015; Mora et al., 2018; Oliva et al., 2014), where it is required together with Dac for the generation of offspring neurons with T4/T5 neuron identity (Apitz and Salecker, 2018). The transcriptional programmes downstream of Ato/Dac conferring T4/T5 neurons with their properties have remained elusive so far.

Here, we perform an RNA interference (RNAi) screen to identify novel transcription factors affecting the acquisition of terminal characters in postmitotic T4/T5 neurons. We use the optomotor response of adult flies as a readout of T4/T5 neuron function and, thus, of proper terminal differentiation. T4/T5 neuron-specific silencing of *SoxN* or *Sox102F*, two members of the Sox family of transcription factors, abolishes the optomotor response in flies, indicative of aberrant T4/T5 neuron maturation. Notably, both transcription factors regulate the acquisition of dendritic and axonal innervation patterns common to all T4/T5 neuron subtypes. We further show that *SoxN* and *Sox102F* regulate the expression of the cell-surface molecule Connectin in all T4/T5 neuron subtypes, although only T4/T5_c,d_ neurons express high Connectin levels in wild-type flies. Finally, we demonstrate that *ato* and *dac* are redundantly required in late-stage dIPC neuroblasts to control SoxN and Sox102F expression in offspring T4/T5 neurons, providing a link between transcription factors previously shown to specify T4/T5 neuron progenitor identity and novel, downstream transcription factors regulating postmitotically morphological properties common to all T4/T5 neurons.

## RESULTS

### Silencing *SoxN* or *Sox102F* in T4/T5 neurons impairs the optomotor response

To find molecular players involved in the terminal differentiation of T4/T5 neurons, we pursued a candidate gene approach focusing on transcription factors revealed to be highly expressed in T4/T5 neurons by a transcriptome analysis (Pankova and Borst, 2016). We performed specific knockdown of these transcription factors in T4/T5 neurons by combining UAS-RNAi effector lines (Dietzl et al., 2007; Perkins et al., 2015) with the *R40E11-Gal4* driver line. *R40E11-Gal4* drives expression in maturing T4/T5 neurons of all subtypes at late third instar (L3) larval stage, and in mature T4/T5_a,b_ neurons at adult stage (Fig. S1A). The optomotor response consists of turning in the direction of a rotating full-field grating and relies on T4/T5 neuron function (Bahl et al., 2013; Maisak et al., 2013). We reasoned that the optomotor response would be affected upon depletion of transcription factors controlling neuronal properties essential for T4/T5 neuron function.

We found that flies expressing either *SoxN-RNAi* or *Sox102F-RNAi* in T4/T5 neurons lacked an optomotor response, similar to flies with blocked synaptic transmission in T4/T5 neurons (Fig. 1D-G) (Bahl et al., 2013; Maisak et al., 2013). Because RNAi might cause off-target effects (Kaya-Copur and Schnorrer, 2016), we confirmed these results by using additional UAS-RNAi transgenes targeting other regions of *SoxN* and *Sox102F* (Fig. S2A-C). Next, we expressed membrane-targeted GFP in T4/T5 neurons using the *R40E11-Gal4* line and performed immunohistochemistry with antibodies recognising SoxN and Sox102F to confirm that both transcription factors were expressed in T4/T5 neurons (Fig. 1H,K). We found strongly reduced levels of SoxN and Sox102F in T4/T5 neurons upon expression of UAS-RNAi transgenes against them, both at late L3 larval and adult stages (Fig. 1H-S, Fig. S2D-G). Together, these results show that sustained knockdown of *SoxN* or *Sox102F* in postmitotic T4/T5 neurons severely impairs the optomotor response.

### SoxN and Sox102F are expressed in all T4/T5 neuron subtypes, but not in their progenitors or in C/T neurons

**Fig. 2.**
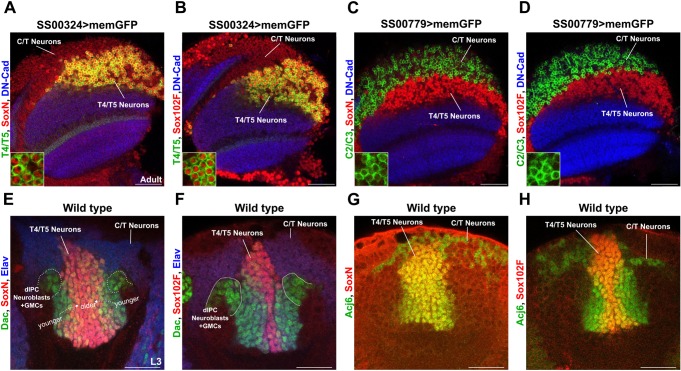
**SoxN and Sox102F are expressed in all T4/T5 neuron subtypes, but not in their progenitors or in C/T neurons.** (A-D) SoxN and Sox102F expression in adult optic lobes with T4/T5 or C2/C3 neurons labelled with memGFP. Neuropils were labelled with anti-DN-Cadherin (DN-Cad). Insets show zoomed views of T4/T5 (A,B) or C2/C3 (C,D) somata. (E,F) SoxN and Sox102F expression in late L3 larval optic lobes after immunostaining against Elav and Dac. Late-stage dIPC neuroblasts and ganglion mother cells (GMCs) are Dac^+^/Elav^−^. All young T4/T5 neurons (closest to dIPC) are Dac^+^/Elav^+^. Older T4/T5_a,b_ neurons are Dac^+^/Elav^+^ whereas older T4/T5_c,d_ neurons are Dac^−^/Elav^+^. (G,H) SoxN and Sox102F expression in late L3 larval optic lobes after immunostaining against Acj6, which labelled somata located in the region occupied by C/T neurons, and T4/T5 somata. Scale bars: 40 µm (A-D); 20 µm (E-H).

To assess further the role of *SoxN* and *Sox102F* during T4/T5 neuron development, we examined their spatial and temporal patterns of expression in more detail. The *SS00324-splitGal4* line labels specifically mature T4/T5 neurons of the four subtypes in the adult (Schilling and Borst, 2015) and all these neurons expressed SoxN and Sox102F (Fig. 2A,B). SoxN and Sox102F were not detected in the region occupied by C/T somata, which were identified with the *SS00779-splitGal4* line (Fig. 2C,D) (Tuthill et al., 2013). Next, we examined the dIPC in late L3 larvae, when it still contains Dac^+^ neuroblasts and Dac^+^ ganglion mother cells producing T4/T5 neurons (Apitz and Salecker, 2015), and found that T4/T5 neuron progenitors lacked SoxN and Sox102F (Fig. 2E,F). In late L3 larvae, all younger, maturing T4/T5 neurons express Dac whereas only older, maturing T4/T5_a,b_ neurons express Dac (Apitz and Salecker, 2018). Both Dac^+^ T4/T5_a,b_ and Dac^−^ T4/T5_c,d_ neurons expressed SoxN and Sox102F (Fig. 2E,F). SoxN and Sox102F were not detected at late L3 larval stage in C/T neurons, which were identified by both the location of their somata and the expression of Abnormal chemosensory jump 6 (Acj6) (Fig. 2G,H) (Apitz and Salecker, 2015). Therefore, SoxN and Sox102F are expressed in immature and mature T4/T5 neurons of all subtypes, yet they are absent in T4/T5 neuron progenitors and developmentally related C/T neurons.

### SoxN-mediated transcriptional activation is required for Sox102F expression in T4/T5 neurons

**Fig. 3.**
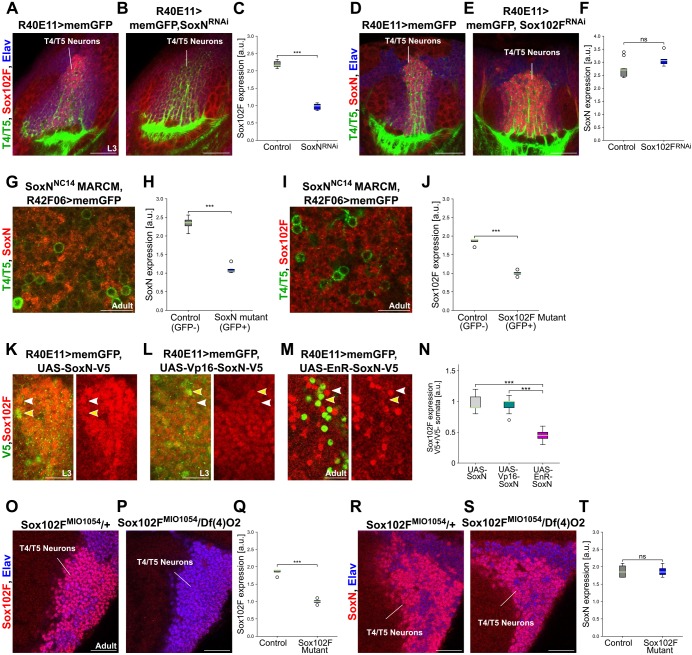
**SoxN-mediated transcriptional activation is required for Sox102F expression in T4/T5 neurons.** (A-C) Sox102F expression in late L3 larval optic lobes with wild-type T4/T5 neurons, and with T4/T5 neurons expressing *SoxN-RNAi*. Quantification is shown in C (control: *n*=6; *SoxN-RNAi*: *n*=7 optic lobes). (D-F) SoxN expression in late L3 larval optic lobes with wild-type T4/T5 neurons, and with T4/T5 neurons expressing *Sox102F-RNAi*. Quantification is shown in F (control: *n*=11; *Sox102F-RNAi*: *n*=5 optic lobes). (G-J) SoxN and Sox102F expression in adult *SoxN^NC14^* heterozygous (GFP^−^) and homozygous (GFP^+^) mutant T4/T5 somata after MARCM. Quantifications are shown in H and J [control (GFP^−^): *n*=6; *SoxN* mutant (GFP^+^): *n*=7 optic lobes]. (K-N) Sox102F expression in T4/T5 neurons upon overexpression of SoxN (wild type), Vp16-SoxN (obligatory activator) or EnR-SoxN (obligatory repressor). The three SoxN versions were epitope-tagged with V5. In each panel, the yellow arrowhead marks a T4 or T5 soma with high V5 levels, and the white arrowhead marks a neighbouring T4 or T5 soma without V5 expression. Quantification is shown in N (*n*=10 pairs of somata per group). (O-T) Sox102F and SoxN expression in adult T4/T5 neurons from controls (*Sox102F^MI1054^/+*) and *Sox102F* mutants (*Sox102F^MI1054^/Df(4)O2*). Quantifications are shown in Q and T (*n*=4 optic lobes per group). ns, not significant (*P*>0.05); ***P<0.001. Scale bars: 20 µm (A,B,D,E,O,P,R,S); 10 µm (G,I,K-M).

In late L3 larvae, younger T4/T5 somata form columns closest to the dIPC whereas older T4/T5 somata are displaced centrally (Apitz and Salecker, 2015). We observed Sox102F and SoxN expression in the most central columns of T4/T5 somata, whereas the T4/T5 somata closer to the dIPC showed only SoxN expression (Fig. 2E-H). This indicates that SoxN is expressed at an earlier time point of T4/T5 neuron maturation than Sox102F, and that *SoxN* might regulate Sox102F expression. Indeed, Sox102F expression was severely reduced in T4/T5 neurons upon *SoxN* knockdown with the *R40E11-Gal4* line (Fig. 3A-C, Fig. S3A-C), and in *SoxN* mutant T4/T5 neurons generated by mosaic analysis with a repressible cell marker (MARCM) (Fig. 3G-J). However, overexpression of a wild-type version, an obligatory activator version, or an obligatory repressor version of SoxN (Bahrampour et al., 2017) did not increase Sox102F levels in T4/T5 somata (Fig. 3K-N). In fact, T4/T5 somata lacked Sox102F when expressing the obligatory repressor version of SoxN (Fig. 3N). Altogether, these results indicate that SoxN-mediated transcriptional activation is required for Sox102F expression in T4/T5 neurons. In contrast, *Sox102F* is dispensable for SoxN expression in T4/T5 neurons, as SoxN levels were unchanged after silencing *Sox102F* in T4/T5 neurons with either RNAi transgenes or the microRNA *mir-263a* (also known as *bft*) (Fig. 3D-F, Fig. S3D-J), which is predicted to target *Sox102F* (according to TargetScanFly, Release 7.2, October 2018) and reduces Sox102F levels (Fig. S3H). In agreement with this, SoxN levels were normal in *Sox102F* mutant flies (Fig. 3O-T), generated by combining the *Sox102F^MI01054^* hypomorphic allele with the *Df(4)O2* deficiency chromosome lacking the *Sox102F* locus (Contreras et al., 2018).

### *SoxN* and *Sox102F* regulate dendritic and axonal development autonomously in T4/T5 neurons, and dendritic development non-autonomously in lobula plate tangential cells

**Fig. 4.**
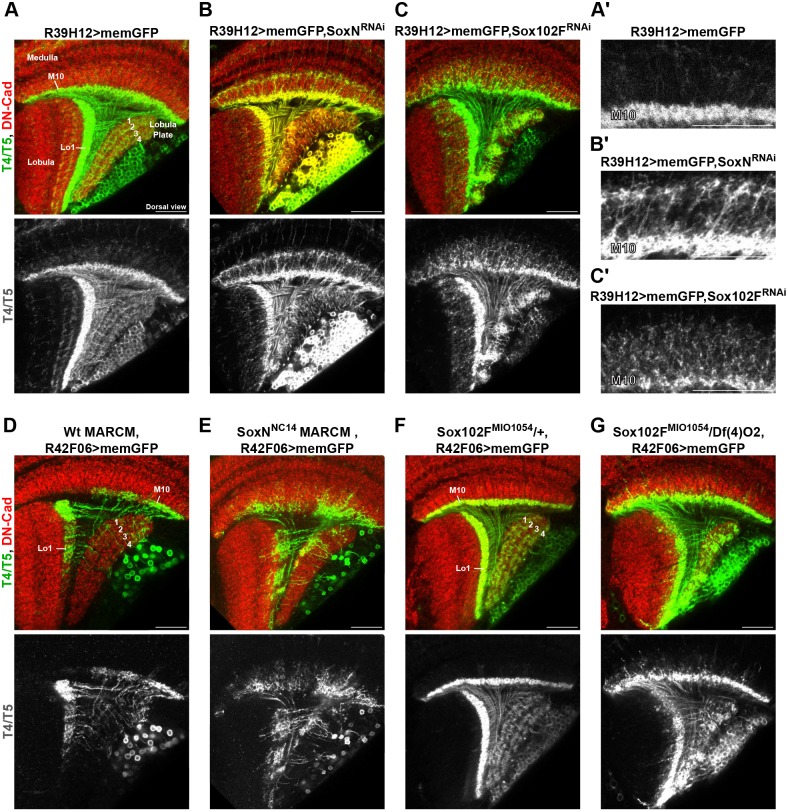
***SoxN* and *Sox102F* control dendritic and axonal morphology in T4/T5 neurons.** (A-C′) Dorsal views of adult optic lobes with wild-type T4/T5 neurons, and with T4/T5 neurons expressing *SoxN-RNAi* or *Sox102F-RNAi*. A′-C′ show detailed views of T4 dendrites from the conditions in A-C. Anterior is to the left. (D,E) Adult optic lobes with wild-type T4/T5 neurons, and *SoxN^NC14^* homozygous mutant T4/T5 neurons generated by MARCM and labelled with the *R42F06-Gal4* line. (F,G) Adult optic lobes with T4/T5 neurons labelled with the *R42F06-Gal4* line in controls (*Sox102F^MI1054^/+*) and in *Sox102F* mutants [*Sox102F^MI1054^/Df(4)O2*]. Scale bars: 20 µm.

Our previous results suggest that *SoxN* and *Sox102F* are part of the terminal differentiation programmes responsible for defining T4/T5 neuron function. One possibility is that *SoxN* and *Sox102F* control morphological properties of T4/T5 neurons. We examined the shape of membrane-targeted GFP-labelled T4/T5 neurons in adult flies in which *SoxN* or *Sox102F* were silenced from late L3 larval stage onwards with either the *R39H12-Gal4* or the *T4/T5-splitGal4* driver line (Fig. S1B,C). Wild-type T4 and T5 dendrites arborised only in medulla layer M10 and in lobula layer Lo1, respectively, and wild-type T4/T5 axons formed four layers in the lobula plate (Fig. 4A,A′, Fig. S4A). In flies expressing *SoxN-RNAi* in all T4/T5 neurons, T4 and T5 dendrites extended into extra medulla and lobula layers, and T4/T5 axons did not form four layers but accumulated predominantly in the most anterior half of the lobula plate (Fig. 4B,B′, Fig. S4B). Very similar defects were observed in *SoxN* mutant T4/T5 neurons (Fig. 4D,E), confirming that these phenotypes are caused by a specific disruption of *SoxN* function. Moreover, T4/T5 neurons expressing an obligatory repressor version of SoxN showed dendritic overgrowth and axons failing to form layers in the lobula plate (Fig. S4G). Upon *Sox102F-RNAi* expression in all T4/T5 neurons, we also observed overgrowth of T4/T5 dendrites and a loss of the layered structure in the lobula plate with T4/T5 axons forming clusters (Fig. 4C,C′, Fig. S4C). The specificity of this phenotype was confirmed by examining T4/T5 neurons in *Sox102F* mutants (Fig. 4F,G) and upon expression of *mir-263a* (Fig. S4D)*.* T4/T5 neurons overexpressing Sox102F also showed dendritic and axonal defects (Fig. S4H-J).

Moreover, lobula plate volume was reduced when *SoxN* or *Sox102F* were silenced in all T4/T5 neurons (Fig. S5A-D), further supporting the conclusion that T4/T5 axons were defective and suggesting that other neurons innervating the lobula plate might be affected. To test this, we labelled lobula plate tangential cells innervating lobula plate layers 1 and 4 with the *VT23749-LexA* line (Mauss et al., 2015) in control flies and in flies expressing *SoxN-RNAi* or *Sox102F-RNAi* in all T4/T5 neurons. When RNAi^+^ T4/T5 axons failed to form layers, dendrites from RNAi^−^ lobula plate tangential cells did not form layers in the lobula plate either (Fig. S5E-G). These results show that *SoxN* and *Sox102F* regulate dendritic and axonal morphology autonomously in T4/T5 neurons, and dendritic morphology non-autonomously in lobula plate tangential cells.

To determine whether the observed phenotypes were caused by developmental defects, we examined wild-type T4/T5 neurons and T4/T5 neurons expressing *SoxN-RNAi* or *Sox102F-RNAi* at late L3 larval stage, and at pupal stages 24 h and 48 h after puparium formation (APF). At late L3 larval stage and at 24 h APF, RNAi^+^ T4/T5 dendrites were indistinguishable from wild-type T4/T5 dendrites (Fig. S6A-F). At 48 h APF, however, we found dendritic overgrowth in RNAi^+^ T4/T5 neurons compared with wild-type T4/T5 neurons. Wild-type T4/T5 axons formed layers in the lobula plate at 48 h APF but not at the earlier stages examined. In contrast, RNAi^+^ T4/T5 axons failed to form distinct layers in the lobula plate at 48 h APF (Fig. S6G-I). Finally, we excluded a transformation of T4/T5 neurons into developmentally related neurons upon *SoxN* and *Sox102F* disruption by examining markers of T4/T5 and C/T neurons. LIM homeobox 1 (Lim1) expression in T4/T5 neurons (Suzuki et al., 2016) was unchanged after *SoxN* and *Sox102F* knockdown, and C/T neuron markers Twin of Eyeless (Toy) (Apitz and Salecker, 2015) and Apterous (Ap) were absent in T4/T5 neurons upon *SoxN-RNAi* and *Sox102F-RNAi* expression (Fig. S7A-L). From these results, we conclude that *SoxN* and *Sox102F* are required in maturing T4/T5 neurons for dendritic and axonal patterning between 24 and 48 h APF stages.

### *SoxN* and *Sox102F* mediate layer-specific innervation of T4/T5 dendrites and axons, and neuropil-specific innervation of T4/T5 axons

**Fig. 5.**
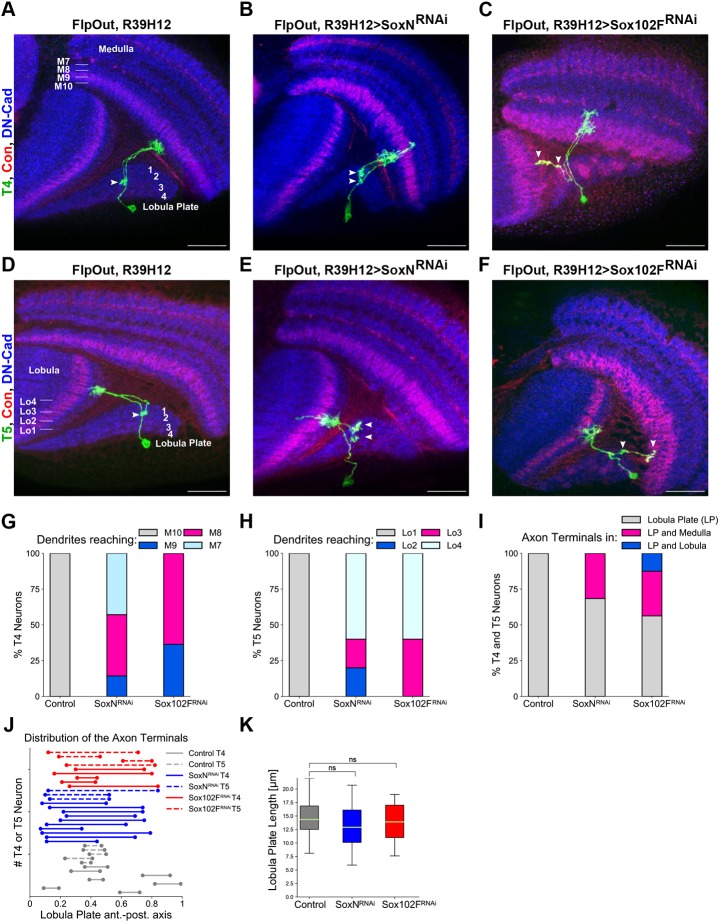
***SoxN* and *Sox102F* mediate layer-specific innervation of T4/T5 dendrites and axons, and neuropil-specific innervation of T4/T5 axons.** (A-F) Adult optic lobes with single-labelled T4 and T5 neurons in control flies, and in flies expressing *SoxN-RNAi* or *Sox102F-RNAi* in all T4/T5 neurons. Neuropil layers were identified after immunostaining against DN-Cad and Connectin. Arrowheads mark the presence of axonal boutons. (G) Percentages of T4 dendrites reaching the medulla layers M10, M9, M8 or M7 in control flies, and in flies expressing *SoxN-RNAi* or *Sox102F-RNAi* in all T4/T5 neurons (control: *n*=9; *SoxN-RNAi*: *n*=14; *Sox102F-RNAi*: *n*=11 neurons). (H) Percentages of T5 dendrites reaching the lobula layers Lo1, Lo2, Lo3 or Lo4 in control flies, and in flies expressing *SoxN-RNAi* or *Sox102F-RNAi* in all T4/T5 neurons (control: *n*=7; *SoxN-RNAi*: *n*=5; *Sox102F-RNAi*: *n*=5 neurons). (I) Percentages of T4 and T5 axons innervating only the lobula plate, co-innervating the lobula plate and the medulla, or co-innervating the lobula plate and the lobula, in control flies and in flies expressing *SoxN-RNAi* or *Sox102F-RNAi* in all T4/T5 neurons (control: *n*=12; *SoxN-RNAi*: *n*=19; *Sox102F-RNAi*: *n*=16 neurons). (J) Extension of lobula plate (normalised) along the anteroposterior axis occupied by T4 and T5 axons in control flies, and in flies expressing *SoxN-RNAi* or *Sox102F-RNAi* in all T4/T5 neurons. 0 and 1 represent the most anterior and the most posterior edges of the lobula plate, respectively. (K) Average lobula plate lengths (absolute values) along the anteroposterior axis at the positions occupied by the analysed axons in J (control: *n*=12, *SoxN-RNAi*: *n*=13, *Sox102F-RNAi*: *n*=9 positions). ns, not significant (*P*>0.05). Scale bars: 20 µm.

To determine unambiguously which aspects of dendritic and axonal patterning are regulated by *SoxN* and *Sox102F*, we analysed individual neurons labelled stochastically by using the Flp-out technique (Nern et al., 2011) after silencing *SoxN* or *Sox102F* in all T4/T5 neurons with the *R39H12-Gal4* line. We defined the neuropil layers innervated by single-labelled T4 and T5 dendrites by staining optic lobes with DN-cadherin (Cadherin-N) and Connectin (Gao et al., 2008; Ngo et al., 2017). In control flies, none of the single-labelled T4 and T5 neurons had dendrites in layers other than medulla layer M10 (Fig. 5A,G) and lobula layer Lo1 (Fig. 5D,H), respectively. After *SoxN* or *Sox102F* silencing in all T4/T5 neurons, in contrast, T4 and T5 dendrites spanned over extra layers of neuropil. T4 dendrites often reached medulla layer M7 in the case of *SoxN* silencing and medulla layer M8 in the case of *Sox102F* silencing, and T5 dendrites often reached lobula layer Lo4 when *SoxN* or *Sox102F* were silenced (Fig. 5A-H). We also found that, in contrast to wild-type T4 and T5 axons, which always innervated exclusively the lobula plate, one-third of T4 and T5 axons co-innervated the lobula plate and the medulla upon *SoxN* or *Sox102F* silencing in all T4/T5 neurons (Fig. 5F,I). On rare occasions, T4 axons co-innervated the lobula plate and the lobula upon *Sox102F* silencing (Fig. 5C,I). Finally, we analysed in more detail those T4 and T5 axons that specifically innervated the lobula plate upon knockdown of *SoxN* or *Sox102F* and compared them with wild-type T4 and T5 axons. Wild-type T4 and T5 axons occupied 13±4% (*n*=12 axons) of the lobula plate along the anteroposterior axis, reflecting layer-specific innervation. Upon *SoxN* or *Sox102F* silencing, T4 and T5 axons occupied 50±13% (*n*=13 axons) or 41±19% (*n*=9 axons) of the lobula plate along the anteroposterior axis, respectively (Fig. 5J). As the length of the lobula plate along the anteroposterior axis was unchanged at the positions occupied by the analysed wild-type and RNAi^+^ T4/T5 axons (Fig. 5K), we concluded that the axons of T4/T5 neurons expressing *SoxN-RNAi* or *Sox102F-RNAi* lack the layer-specific innervation characteristic of wild-type T4/T5 neurons. These results demonstrate a requirement of *SoxN* and *Sox102F* for the development of the layer-specific innervation of T4/T5 dendrites and axons, and the neuropil-specific innervation of T4/T5 axons.

### *SoxN* and *Sox102F* control the layer specificity of dendrites and axons autonomously in different T4/T5 neuron subtypes

**Fig. 6.**
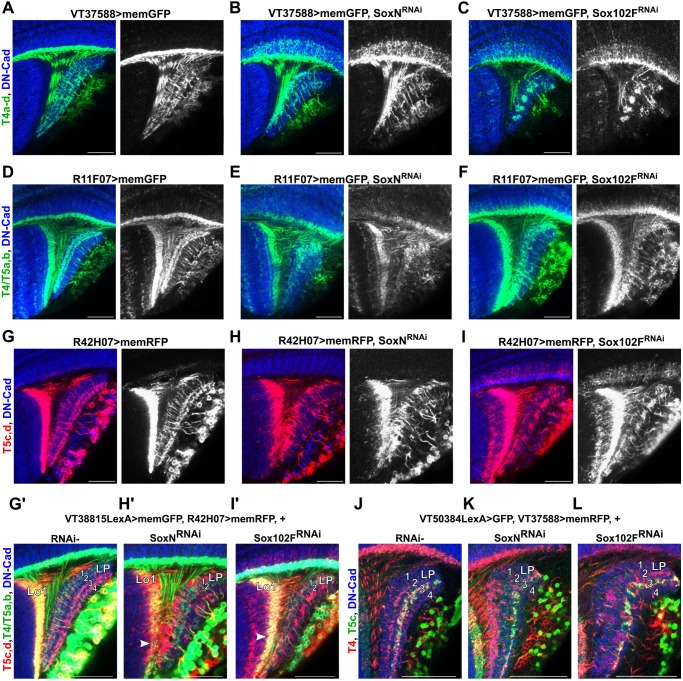
***SoxN* and *Sox102F* regulate the layer specificity of dendrites and axons autonomously in different T4/T5 neuron subtypes.** (A-C) Adult optic lobes with wild-type T4_a-d_ neurons, and with T4_a-d_ neurons expressing *SoxN-RNAi* or *Sox102F-RNAi.* (D-F) Adult optic lobes with wild-type T4/T5_a,b_ neurons, and with T4/T5_a,b_ neurons expressing *SoxN-RNAi* or *Sox102F-RNAi*. (G-I′) Adult optic lobes with wild-type T5_c,d_ neurons, and with T5_c,d_ neurons expressing *SoxN-RNAi* or *Sox102F-RNAi*. RNAi^−^ T4/T5_a,b_ neurons are shown in G′-H′. Arrowheads indicate T5_a,b_ dendrites restricted to lobula layer Lo1 in the presence of defective, RNAi^+^ T5_c,d_ dendrites. LP, lobula plate. (J-L) Detailed views of the lobula plate showing T5_c_ axons in the presence of wild-type T4 axons, and in the presence of T4 axons expressing *SoxN-RNAi* or *Sox102F-RNAi*. Scale bars: 20 µm.

Next, we investigated whether *SoxN* and *Sox102F* control neuronal morphology in every T4/T5 neuron subtype in a similar and autonomous manner. To this end, we first silenced *SoxN* or *Sox102F* in specific T4/T5 neuron subsets with the *VT37588-Gal4* (Maisak et al., 2013), *R11F07-Gal4* and *R42H07-Gal4* (Maisak et al., 2013) lines. These lines labelled, respectively, T4_a-d,_ T4/T5_a,b_ and T5_c,d_ neurons in the adult, and drive gene expression in T4/T5 neuron subsets already at late L3 larval or early pupal stages (Fig. S1D-F). Knockdown of *SoxN* or *Sox102F* in T4_a-d_ and T5_c,d_ neurons caused severe dendritic and axonal defects (Fig. 6A-C,G-I), which resembled the defects observed upon their knockdown using lines driving expression in all T4/T5 neurons (Fig. 4A-C). Defects in layer specificity of dendrites and axons were also observed upon *SoxN* or *Sox102F* silencing in T4/T5_a,b_ neurons (Fig. 6D-F), although these defects were less pronounced than those observed upon silencing them in T4_a-d_ and T5_c,d_ neurons.

Next, we examined the morphology of RNAi- T4/T5 neurons in the presence of RNAi^+^, defective T4/T5 neurons from other subtypes. The dendrites of RNAi- T5_a,b_ neurons, labelled with the *VT38815-LexA* line, did not reach other layers than lobula layer Lo1 in the presence of RNAi^+^ T5_c,d_ dendrites with overgrowth. Moreover, the axons of RNAi- T5_a,b_ neurons were undistinguishable from control T5_a,b_ axons when RNAi^+^, defective T5_c,d_ axons were present (Fig. 6G-I′). In line with this, RNAi- T5_c_ axons, marked with the *VT50384-LexA* line (Haag et al., 2016), terminated in lobula plate layer 3 in spite of the presence of RNAi^+^, defective T4 axons (Fig. 6J-L). Altogether, these results indicate that *SoxN* and *Sox102F* control the layer specificity of dendrites and axons autonomously in each T4/T5 neuron subtype.

### *SoxN* and *Sox102F* are required for the regulation of Connectin levels in T4/T5 neurons

**Fig. 7.**
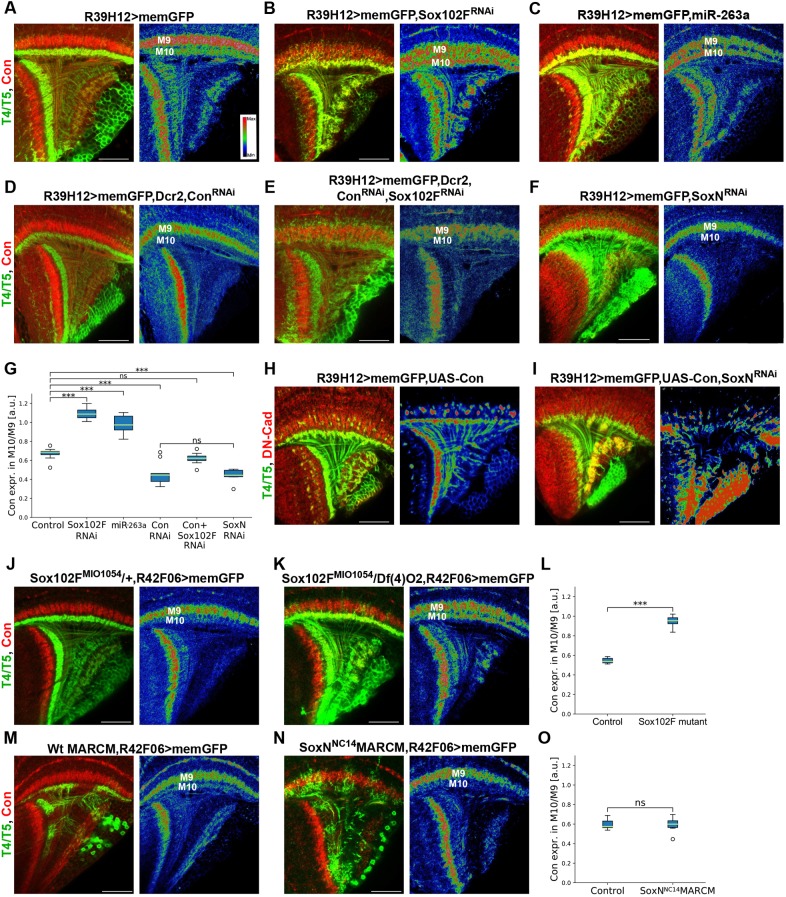
***SoxN* and *Sox102F* are required for the regulation of Connectin levels in T4/T5 neurons.** (A-G) Connectin expression in adult optic lobes with wild-type T4/T5 neurons, and with T4/T5 neurons expressing *Sox102F-RNAi*, *mir-263a*, *Connectin-RNAi*, *Sox102F-RNAi* and *Connectin-RNAi*, or *SoxN-RNAi*. The right panels show Connectin signals colour-coded for intensity. Ratios of Connectin signal in medulla layer M10 to Connectin signal in medulla layer M9 are shown in G (*n*=8-11 optic lobes per group). (H,I) Adult optic lobes with T4/T5 neurons overexpressing *UAS-Connectin* alone or with *SoxN-RNAi*. Overexpression of Connectin induces the formation of axon clusters. (J-L) Connectin expression in adult optic lobes with T4/T5 neurons labelled with the *R42F06-Gal4* line in controls (*Sox102F^MI1054^/+*) and in *Sox102F* mutants [*Sox102F^MI1054^/Df(4)O2*]. Quantification is shown in L (control: *n*=7; *Sox102F* mutant: *n*=9 optic lobes). (M-O) Connectin expression in adult optic lobes with wild-type T4/T5 neurons, and *SoxN^NC14^* homozygous mutant T4/T5 neurons generated by MARCM and labelled with the *R42F06-Gal4* line. Quantification in shown in O (control: *n*=7; *SoxN^NC14^* MARCM: *n*=14 optic lobes). In H-N, right-hand panels show Connectin signals colour-coded for intensity. ns, not significant (*P*>0.05); ***P<0.001. Scale bars: 20 µm.

We noticed an increase in Connectin levels in the neuropil layers occupied by T4/T5 dendrites upon *Sox102F* silencing in all T4/T5 neurons, and in *Sox102F* mutants (Fig. 7A-C,G,J-L). To determine whether this is caused by a non-cell-autonomous mechanism, such as ectopic innervation of these layers by adjacent Connectin-expressing neurons, we co-expressed *Sox102F-RNAi* with a validated *Connectin-RNAi* (Fig. 7D,G) in all T4/T5 neurons. In this condition, Connectin levels in medulla layer M10 were comparable to those in controls (Fig. 7E,G), demonstrating that *Sox102F* is required cell-autonomously in T4/T5 neurons for repressing Connectin expression.

In wild-type flies, Connectin signal is higher in layers 3 and 4 than in layers 1 and 2 of the lobula plate (Fig. 7A) (Gao et al., 2008), suggesting a higher Connectin expression in T4/T5_c,d_ than in T4/T5_a,b_ neurons. A recent transcriptome study has indeed revealed that T5_c,d_ neurons express higher Connectin than T5_a,b_ neurons (Davis et al., 2018 preprint). T4/T5 neuron subtype-specific expression of other transcription factors controlling Connectin expression might influence the capacity of distinct T4/T5 neuron subtypes to upregulate Connectin upon *Sox102F* knockdown. This is not the case, however, as *Sox102F-RNAi* expression in T4/T5_a,b_, T5_c,d_ or T4_c,d_ neurons also resulted in Connectin upregulation (Fig. S8A-I).

Because Sox102F expression in T4/T5 neurons requires *SoxN*, we expected *SoxN* and *Sox102F* loss of function to upregulate Connectin levels similarly. However, Connectin levels did not increase in T4/T5 neurons expressing *SoxN-RNA*i or in *SoxN* mutant T4/T5 neurons (Fig. 7F,G,M-O). We excluded that this is caused by a transformation of T4/T5_c,d_ (high Connectin) into T4/T5_a,b_ (low Connectin) neurons because the proportion of Dac^+^/Omb^−^ (T4/T5_a,b_) and Dac^−^/Omb^+^ (T4/T5_c,d_) neurons upon *SoxN* silencing was the same as in controls (Fig. S7M-O). Collectively, these observations are consistent with *SoxN* regulating Connectin expression in all T4/T5 neuron subtypes through two pathways with opposing effects. Firstly, *SoxN* is required for Sox102F expression, which in turn is necessary for repressing Connectin expression. Secondly, *SoxN* is required for Connectin expression in a *Sox102F*-independent manner. Finally, we attempted to support this model by performing overexpression experiments. However, Sox102F or SoxN overexpression was not sufficient to downregulate or upregulate, respectively, Connectin expression in T4/T5 neurons (Fig. S8J-O). This might be due to wild-type expression levels of proteins required for SoxN and Sox102F transcriptional activity in T4/T5 neurons (She and Yang, 2015).

### *ato* and *dac* are redundantly required in late-stage dIPC neuroblasts for SoxN and Sox102F expression in offspring neurons

**Fig. 8.**
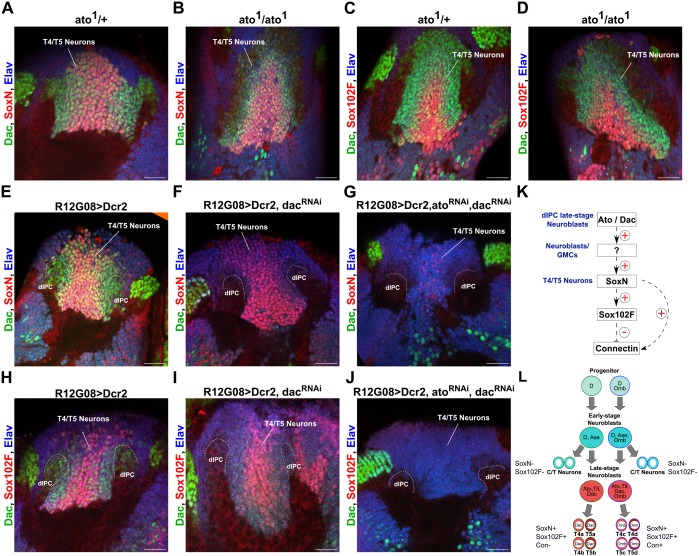
***ato* and *dac* are redundantly required in late-stage dIPC neuroblasts to control SoxN and Sox102F expression in offspring neurons.** (A-D) SoxN and Sox102F expression in prepupal optic lobes from *ato^1^* heterozygous and homozygous mutants. Neuronal somata were marked with anti-Elav. T4/T5 somata were labelled with anti-Dac. (E-J) SoxN and Sox102F expression in prepupal optic lobes with wild-type, late-stage dIPC neuroblasts, and with late-stage dIPC neuroblasts expressing *Dac-RNAi* alone or together with *Ato-RNAi*. Dac signal was absent in the dIPC upon *Dac-RNAi* expression with the *R12G08-Gal4*. (K) Summary of genetic interactions between *ato*/*dac*, *SoxN*, *Sox102F* and *Connectin* revealed in this study. (L) Summary of transcription factors expressed in early- and late-stage dIPC neuroblasts, and in postmitotic T4/T5 neurons revealed by this and a previous study (Apitz and Salecker, 2018). Differential expression of Connectin between T4/T5 neuron subtypes is also depicted. Scale bars: 20 µm.

Loss of the transcription factor Ato in late-stage dIPC neuroblasts, which normally produce T4/T5 neurons, results in offspring neurons with fasciculation problems, dendritic overgrowth (Oliva et al., 2014) and a global downregulation of genes involved in neuronal differentiation (Mora et al., 2018). Loss of the transcription factor Dac in T4/T5 neuron progenitors results in offspring neurons with dendrites that overgrow into medulla layer M9 and more distal layers, and axons that lack neuropil-specific innervation (Apitz and Salecker, 2018). These defects resemble the anatomical phenotypes we reported upon silencing *SoxN* or *Sox102F* in T4/T5 neurons. Ato and/or Dac might start transcriptional programmes in late-stage dIPC neuroblasts that eventually control SoxN and Sox102F expression in postmitotic T4/T5 neurons. To test this, we analysed SoxN and Sox102F expression in T4/T5 neurons in *ato* mutants (Fig. 8A-D) (Jarman et al., 1994) and in flies expressing a validated *dac-RNAi* in the dIPC with the *R12G08-Gal4* line (Fig. 8E,F,H,I) (Apitz and Salecker, 2018). In both experiments, SoxN and Sox102F were detected in the region occupied by T4/T5 somata at prepupal stages, demonstrating that disrupting *ato* or *dac* individually in late-stage dIPC neuroblasts does not abolish SoxN and Sox102F expression in offspring T4/T5 neurons.

*ato* and *dac* in late-stage dIPC neuroblasts might redundantly control SoxN and Sox102F expression in T4/T5 neurons, leading to one transcription factor compensating for the silencing of the other. In fact, only after simultaneous silencing of *ato* and *dac* in late-stage dIPC neuroblasts, do offspring neurons fail to acquire T4/T5 neuron morphologies, indicating a redundant role of *ato* and *dac* in the control of T4/T5 neuron identity (Apitz and Salecker, 2018). This model predicts that removing simultaneously *ato* and *dac* in late-stage dIPC neuroblasts should remove factors controlling the maturation of T4/T5 neurons, such as SoxN and Sox102F. We co-expressed validated *ato-RNAi* and *dac-RNAi* in the dIPC with the *R12G08-Gal4* line (Apitz and Salecker, 2018) and found that most of the neurons in the region normally occupied by T4/T5 somata at prepupal stages lacked SoxN and Sox102F expression (Fig. 8G,J). The remaining SoxN^+^ and Sox102F^+^ neurons might be due to incomplete RNAi knockdown. These results demonstrate that *ato* and *dac* are redundantly required in late-stage dIPC neuroblasts for SoxN and Sox102F expression in offspring neurons.

## DISCUSSION

As neurons are generated during development, they acquire a rich and diverse repertoire of morphological and physiological properties in order to form functional neural circuits. T4/T5 neurons represent a very interesting model for understanding this process. All T4/T5 neurons share a set of terminal characteristics, such as dendrites with a stereotyped size and arborisation in single layers of neuropil, and axons terminating in one of the four layers of the lobula plate. These properties are essential for their function as local motion sensors and to communicate with downstream neurons. At the same time, four subtypes of T4/T5 neurons exist with differences in directional tuning and the layer of the lobula plate innervated by their axons, the latter defining the specific postsynaptic partners of each subtype. Here, we show that the acquisition of morphological properties common to all T4/T5 neuron subtypes is controlled by the postmitotic transcription factors SoxN and Sox102F. Moreover, the two transcription factors appear to play a permissive role in the emergence of subtype-specific properties in T4/T5 neurons. Therefore, SoxN and Sox102F represent two core components of the programmes controlling the maturation of postmitotic T4/T5 neurons and that act downstream of the identity programmes initiated by Ato and Dac in T4/T5 neuron progenitors (Apitz and Salecker, 2018). In conjunction with other recent studies (Apitz and Salecker, 2015, 2018; Mora et al., 2018; Pinto-Teixeira et al., 2018), our work provides a basis for understanding precisely how T4/T5 neurons acquire their properties during development.

Temporal and spatial transcription factors specify neuroblast identity in the developing *Drosophila* optic lobe (Apitz and Salecker, 2015, 2018; Erclik et al., 2017; Li et al., 2013; Suzuki et al., 2013). How do these transcription factors regulate the acquisition of properties defining identity and function in offspring neurons? Homothorax (Hth) is one of the temporal transcription factors expressed in medulla neuroblasts. Hth expression is maintained in the progeny of Hth^+^ neuroblasts, including Mi1 neurons, where it controls neuronal morphology in part by regulating Brain-specific homeobox (Bsh) and DN-cadherin expression (Hasegawa et al., 2013, 2011; Li et al., 2013; Suzuki et al., 2013). Other temporal transcription factors expressed in medulla neuroblasts, such as Klumpfuss (Klu), are not maintained in offspring neurons and are thought to control neuronal terminal differentiation through an intermediate tier of transcription factors (Li et al., 2013; Suzuki et al., 2013). These two complementary mechanisms for conveying identity information from neuroblasts to neurons also occur during T4/T5 neuron development. Omb is initially expressed in a subset of spatially patterned T4/T5 neuron progenitors and is maintained in their offspring T4/T5_c,d_ neurons, where it controls subtype-specific properties (Apitz and Salecker, 2018). In contrast, Ato is transiently expressed in T4/T5 neuron progenitors. There, together with Dac, it initiates transcriptional programmes important for T4/T5 neuron specification (Apitz and Salecker, 2018). The output of these programmes comprises, at least, SoxN and Sox102F expression in postmitotic T4/T5 neurons. Future studies will be required to identify the transcriptional cascade linking Ato/Dac with SoxN/Sox102F, and other transcription factors controlling the terminal differentiation of postimitotic T4/T5 neurons. Neuron type-specific expression of transcription factors restricted to postmitotic stages also generates neuronal diversity in nematodes and in mammals (Hobert, 2016; Lodato and Arlotta, 2015). Therefore, we expect that our findings will help to understand how the expression of this type of transcription factors is selected during development in invertebrates and vertebrates.

T4/T5 neurons require *SoxN* and *Sox102F* function in order to establish their specific innervation patterns. In particular, our results indicate that *SoxN* and *Sox102F* are required for maintaining T4/T5 dendrites and axons within single neuropil layers during neuronal maturation, rather than for guiding them towards the correct target layers (Fig. 5, Fig. S6). In order to understand the cellular and molecular mechanisms preventing the overgrowth of T4/T5 dendrites and axons into extra neuropil layers, future studies will be needed to characterise in detail the development of both wild-type T4/T5 neurons and T4/T5 neurons with *SoxN* or *Sox102F* loss of function, and to identify the direct targets of SoxN and Sox102F in T4/T5 neurons. These studies will also shed light on the redundant and distinct functions of *SoxN* and *Sox102F* during T4/T5 neuron development.

*SoxN* and *Sox102F* control dendritic and axonal patterning in every T4/T5 neuron subtype. However, we found that silencing *SoxN* or *Sox102F* in T4/T5_a,b_ caused milder phenotypes than in T4/T5_c,d_ neurons. This might result from different activities of these transcription factors in distinct T4/T5 neuron subtypes upon subtype-specific post-translational modifications or subtype-specific expression of transcriptional co-regulators (Savare et al., 2005; She and Yang, 2015). Alternatively, SoxN and Sox102F transcriptional activities might be equivalent in all T4/T5 neuron subtypes. In this scenario, the differences observed between T4/T5 neuron subtypes upon *SoxN* or *Sox102F* knockdown could result from disparate expression levels of target genes that are dysregulated in an equivalent manner but have different basal expression levels between subtypes. In line with this, T4/T5_a,b_ and T4/T5_c,d_ neurons with knockdown of *Sox102F* seem to express moderate and high levels of Connectin, respectively (Fig. S8B,E). This is likely the result of combining a similar upregulation of Connectin in T4/T5_a,b_ and T4/T5_c,d_ neurons with T4/T5_c,d_ expressing higher basal levels of Connectin than T4/T5_a,b_ neurons. As Connectin functions as a homophilic cell-adhesion molecule (Nose et al., 1997; Raghavan and White, 1997), high Connectin levels might be the cause of T4/T5 axons forming clusters upon Sox*102F* silencing. In agreement with this, Connectin overexpression in wild-type T4/T5 neurons or in T4/T5 neurons with *SoxN* silenced induced the formation of axon clusters (Fig. 7H,I). However, knockdown of *Connectin* in T4/T5 neurons with *Sox102F* silenced did not rescue the formation of axon clusters (Fig. 7E). Therefore, one possibility is that other cell-surface molecules dysregulated upon *Sox102F* silencing play redundant roles with Connectin in the generation of this phenotype. Furthermore, *Connectin* knockdown in wild-type T4/T5 neurons did not prevent their axons from forming layers in the lobula plate (Fig. 7D). The role of the differential expression of Connectin between T4/T5 neuron subtypes needs further investigation. Finally, the disparity in the basal levels of Connectin between T4/T5 neuron subtypes appears to emerge from its tight regulation by transcription factors present in all T4/T5 neuron subtypes, such as SoxN and Sox102F, and transcription factors expressed in a subtype-specific manner, such as Omb (Fig. 8K,L) (Apitz and Salecker, 2018). Future studies should explore whether the same holds true for other molecular players controlling subtype-specific morphological properties in T4/T5 neurons. We envision T4/T5 neurons as a powerful system for improving our understanding of how the acquisition of properties common to a neuronal population and the acquisition of subtype-specific characters within this population are coordinated at the transcriptional level.

In addition, some of our findings bring insight into the development of motion vision circuits in *Drosophila* and of layered neural circuits in general. When we disrupted the dendrites and axons of specific T4/T5 neuron subtypes by silencing *SoxN* or *Sox102F*, the dendrites and axons from other T4/T5 neuron subtypes were unaffected. Therefore, SoxN and Sox102F act autonomously in distinct T4/T5 neuron subtypes to ensure the layer specificity of their dendrites and axons. In contrast, when T4/T5 axons failed to form distinct layers in the lobula plate, the dendrites of their postsynaptic partners also failed to form layers, suggesting that T4/T5 neurons play an instructive role in lobula plate patterning. The finding that *SoxN* and *Sox102F* function in T4/T5 neurons is non-autonomously required for the layer specificity of lobula plate tangential cells supports a recently proposed model describing layer formation as a stepwise process relying on transcription factors that restrict neurons to specific layers in a cell-intrinsic manner, and recruit other circuit components in a cell-extrinsic manner (Peng et al., 2018).

Finally, our finding that *SoxN* plays a role in the terminal differentiation of T4/T5 neurons is in agreement with previous studies that implicated *SoxN* both in the regulation of terminal differentiation genes (Ferrero et al., 2014; Girard et al., 2006) and in the control of axonal patterning in *Drosophila* embryonic neurons (Girard et al., 2006; Overton et al., 2002). *SoxN* and its mammalian orthologues, the SoxB1 family genes, have conserved roles in neural stem cell development (Neriec and Desplan, 2014; She and Yang, 2015). Whether mammalian SoxB1 transcription factors regulate the terminal differentiation of neurons is unclear. In mammals, members of the SoxD family are well known for controlling neuronal terminal differentiation (She and Yang, 2015). A previous study showed that neuronal silencing of *Sox102F*, the only *Drosophila* orthologue of the SoxD family (Phochanukul and Russell, 2010), leads to abnormal neuronal development and behavioural impairment (Li et al., 2017). However, this study did not identify the specific developmental processes affected. Our work, in agreement with a recent study (Contreras et al., 2018), demonstrates a conserved role of *Sox102F* in controlling the terminal differentiation of neurons. Moreover, our observations suggest that one of the roles of *Sox102F* in T4/T5 neurons is to prevent the acquisition of morphological traits characteristic of developmentally related T2/T3 neurons, such as dendritic arborisation in medulla layer M9 and axons in the lobula. In agreement with this, *Sox102F* negatively regulates Connectin expression in T4/T5 neurons, which is highly expressed in T3 neurons (Konstantinides et al., 2018). *Sox5*, a member of the mammalian SoxD family, regulates postmitotically the molecular identity and connectivity of early-born corticofugal neurons by repressing the expression of genes characteristic of late-born corticofugal neurons (Kwan et al., 2008; Lai et al., 2008). Therefore, SoxD-mediated transcriptional repression in postmitotic neurons might represent a conserved mechanism for controlling neuronal identities in vertebrates and invertebrates.

## MATERIALS AND METHODS

### Fly strains

Flies were raised at 25°C and 60% humidity on standard cornmeal agar medium at 12 h light/dark cycle, except for RNAi experiments, in which offspring were shifted from 25°C to 29°C at first larval instar stage. At larval and pupal stages, female and male brains were analysed. At adult stages, only female brains were analysed. The following fly strains were used as driver lines (BL# strains are from Bloomington *Drosophila* Stock Center): *R40E11-Gal4* (BL#48140), *SS00324-splitGal4 (R59E0-AD attP40; R42F06-DBD attP2)* (Schilling and Borst, 2015), *SS00779-splitGal4 (R20C11-AD attP40; R48D11-DBD attP2)* (Tuthill et al., 2013), *R39H12-Gal4* (BL#50071), *VT37588-Gal4* (Maisak et al., 2013), *R11F07-Gal4* (BL#39414), *R42H07-Gal4* (BL#50172), *R12G08-Gal4* (BL#47855), *VT23749-LexA* (Mauss et al., 2015), *VT38815-LexA* and *VT50384-LexA* (Haag et al., 2016). The *T4/T5-splitGal4* driver line was generated by combining the *R41G11-AD* (BL#71050) and *R39H12-DBD* (BL#69444) hemidriver lines (Dionne et al., 2018). The *T4_c,d_-splitGal4* driver line was generated by combining the *VT16255-AD* (BL#75205) and *VT37588-DBD* (BL#75793) hemidriver lines (Tirian and Dickson, 2017 preprint). The following fly strains were used as reporter lines: *UAS-myr::GFP* (BL#32198), *UAS-mCD8::GFP* (BL#32188), *UAS-CD4-tdGFP* (BL#35836), *UAS-myr::tdTomato* (BL#32222), *UAS-mCD8::RFP*, *LexAop-mCD8::GFP* (BL#32229) and *LexAop-GCaMP6m* (BL#44275). To label individual neurons stochastically, we combined Gal4 driver lines with the following lines: *R57C10-Flp2::PEST* (Nern et al., 2015) and *UAS-(FRT.stop)myr::GFP* (Nern et al., 2011). For knockdown experiments, we used the following lines: *UAS-Dcr2* (BL#24646, BL#24650 and BL#24651), *UAS-GFP-RNAi* (BL#41553), *UAS-SoxN-RNAi* (Vienna *Drosophila* Stock Center, shRNA-330056), *UAS-SoxN-RNAi2* (BL#25996), *UAS-Sox102F-RNAi* (Vienna *Drosophila* Stock Center, shRNA-330016), *UAS-Sox102F-RNAi2* (BL#26220), *UAS-mir-263a* (BL#59894), *UAS-Connectin-RNAi* (BL#28967), *UAS-dac-RNAi* (Vienna *Drosophila* Stock Center, KK106040) and *UAS-ato-RNAi* (BL#26316). For overexpression experiments, we used the following lines: *UAS-SoxN-V5* (wild-type version of SoxN), *UAS-Vp16-SoxN-V5* (obligatory activator version of SoxN), *UAS-EnR-SoxN-V5* (obligatory repressor version of SoxN) (provided by S. Thor, Linköping University, Sweden), *UAS-Sox102F* and *UAS-Connectin* (this study). The *UAS-shi^ts^* line (BL#66600) was used to block synaptic transmission at elevated temperatures (Kitamoto, 2001). We also used *Canton-S* (BL#64349) as a wild-type strain, *Sox102F^MI01054^* flies (BL#32729), *Df(4)O2* flies (BL#7084), and the *ato^1^* mutant strain (a gift from B. A. Hassan, ICM – Hôpital Pitié Salpêtrière, Paris). MARCM experiments were performed by crossing *hs-Flp UAS-mCD8::GFP; tub-Gal80 FRT40A; R42F06-Gal4/TM6* flies (a gift from F. Pinto-Teixeira, New York University, USA) with *FRT40A* flies (a gift from I. Salecker, Francis Crick Institute, UK) or *SoxN^NC14^ FRT40A/CyO* flies [generated by us after recombining *FRT40A* with the *SoxN^NC14^* mutant allele (BL#9937)]. Second and third instar larvae resulting from these crosses were heat shocked for 120 min in a 37°C water bath.

The *UAS-Sox102F* and *UAS-Connectin* strains were generated as follows: *Sox102F* and *Connectin* DNAs were produced by gene synthesis (Genewiz) based on the FlyBase CDS sequences FBpp0100057 and FBpp0073231, respectively, and subsequently cloned into *Xho*I-*Xba*I sites of *pJFRC7-20XUAS-IVS-mCD8::GFP* (Pfeiffer et al., 2010; Addgene plasmid #26220, deposited by Gerald Rubin), after removal of the *mCD8::GFP* cassette. The resulting *UAS-Sox102F* and *UAS-Connectin* plasmids were injected into the *su(Hw)attP1* landing site strain BL#34760 and the *VK05* landing site strain BL#9725, respectively, for PhiC31 integrase-mediated transgenesis (BestGene).

### Behavioural assay and analysis

We cold-anaesthetised adult flies before the experiment and glued head, thorax and wings to a needle with bonding glue (Sinfony Opaque Dentin) under blue LED light (440 nm). Afterwards, we positioned animals on an air-suspended polyurethane ball. A virtual environment was projected onto three high-definition screens that collectively spanned 270° (along the vertical axis) and 114° (along the horizontal) of the fly eye's visual field. This gave an approximate spatial resolution of below 0.1°. We used six such set-ups for recording fly locomotion as described previously (Bahl et al., 2013). Two set-ups displayed stimuli at a refresh frequency of 120 Hz; on four set-ups, the frequency was 144 Hz. All monitors were equilibrated in brightness and contrast. Within the immediate area surrounding the fly, we controlled temperature using a custom-built closed-loop thermoregulation system. For the first 5 min, temperature was kept at 25°C and then raised to 34°C within 10 min. We used an optomotor stimulus similar to previous studies (Bahl et al., 2013). Flies were presented with a stationary square wave grating that had a spatial wavelength of 45° in visual angle and a Michelson contrast of 50%. Each individual trial lasted 8 s. Between 2 s and 6 s, the pattern travelled at a fixed velocity of 50°/s (corresponding to a temporal contrast frequency of 2 Hz) in either clockwise or counterclockwise direction. We repeated the stimulus 55 times per fly. The pattern was rendered in real-time using Panda3D, an open source game engine, and Python 2.7.

Data were processed as described previously (Ammer et al., 2015). Tracking data from optical sensors were processed at 4 kHz, read out via a USB interface, and recorded by a computer at 100 Hz. This allowed real-time calculation of the instantaneous rotation axis of the sphere. Rotation traces were re-sampled to 20 Hz for further processing and fed through a first-order low-pass filter with a time constant of 100 ms. We manually selected 30 consecutive trials that fulfilled the following criteria. First, the average turning tendency of the fly was roughly zero. Second, the mean forward velocity of the fly was at least 5* *mm/s, indicating visual responsiveness. For further processing, we subtracted responses for the two symmetrical pattern directions to reduce the magnitude of residual walking asymmetries. We then took the mean across trials. For statistical purposes, we calculated the optomotor response of each fly as the average of the turning response between 4 s and 6 s. All data analysis was performed using Python 2.7 and the NumPy library.

### Antibodies and immunolabelling

Primary antibodies used in this study were: rabbit anti-GFP (1:600, Biolabs, TP401), mouse anti-GFP (1:600, Sigma-Aldrich, G6539), chicken anti-GFP (1:600, ThermoFisher, A10262). rat anti-DN-Cadherin (1:50, Developmental Studies Hybridoma Bank, AB528121), mouse anti-Connectin (1:50, Developmental Studies Hybridoma Bank, AB10660830), rabbit anti-DsRed (1:1000, Clontech, 632496), mouse anti-Dachshund (1:20, Developmental Studies Hybridoma Bank, AB528190), rat anti-Elav (1:50, Developmental Studies Hybridoma Bank, Rat-Elav-7E8A10), mouse anti-Acj6 (1:50, Developmental Studies Hybridoma Bank, AB528067), rabbit anti-SoxN (1:300, a gift from S. Russell; Ferrero et al., 2014), rabbit anti-Sox102F (1:300), rabbit anti-Lim1 (1:500), rabbit anti-Toy (1:1000), rabbit anti-Ap (1:200) (gifts from C. Desplan, New York University, USA) and rabbit anti-Omb (1:400, a gift from G. Pflugfelder, Johannes Gutenberg-University, Mainz, Germany). Secondary antibodies used in this study were: Alexa Fluor 488-conjugated goat anti-rabbit (Invitrogen, A11034), Alexa Fluor 488-conjugated goat anti-mouse (Thermo Fisher, A28175), Alexa Fluor 488-conjugated goat anti-rat (Invitrogen, A11006), Alexa Fluor 488-conjugated donkey anti-chicken (Jackson ImmunoResearch, 703-545-155), Alexa Fluor 568-conjugated goat anti-rabbit (Life Technologies, A11011), Alexa Fluor 568-conjugated goat anti-mouse (Invitrogen, A11004), ATTO 647N-conjugated goat anti-mouse (Rockland, 610-156-040) and Alexa Fluor 647-conjugated goat anti-rat (Life Technologies, A21247) (all used at 1:500).

For immunolabelling, brains were dissected in cold PBS and fixed in 4% formaldehyde (containing 0.3% Triton X-100) at room temperature for 24 (adult) or 15 (larval and pupal) min. Afterwards, they were washed three times with PBT (PBS containing 0.3% Triton X-100) and blocked with 10% normal goat serum in PBT at room temperature for 2 h. Brains were incubated with primary antibodies diluted in PBT containing 5% normal goat serum for 24-48 h at 4°C. After washing five times with PBT, brains were incubated with secondary antibodies diluted in PBT containing 5% normal goat serum for 24-48 h at 4°C. After washing five times with PBT and one time with PBS, brains were mounted in Gold Antifade Mountant (Thermo Fisher).

### Imaging and quantification

Imaging was performed with a Leica SP8 laser scanning confocal microscope equipped with 488-, 561- and 633-nm lasers, and using a 63× objective. Image processing and quantitative analyses were performed with the Fiji software package (Schindelin et al., 2012).

Relative expression levels of SoxN or Sox102F in T4/T5 neurons were quantified as follows: for each optic lobe, we measured the mean grey values (anti-SoxN or anti-Sox102F channel) of either at least 15 manually segmented T4/T5 somata (in Fig. 1N-S and Fig. 3G-J) or the region occupied by T4/T5 somata (Fig. 1H-M and Fig. 3A-F,O-T) across several optical sections. Afterwards, we obtained the average of these values and divided it by the average of mean grey values (anti-SoxN or anti-Sox102F channel) measured in the region occupied by C/T somata across several optical sections. To assess changes in Sox102F expression in T4/T5 neurons upon overexpression of different V5-tagged versions of SoxN (Fig. 3K-N), we measured the mean grey value (anti-Sox102F channel) of a manually segmented T4/T5 soma overexpressing SoxN (high anti-V5 signal), and divided it by the mean grey value (anti-Sox102F channel) of a neighbouring T4/T5 soma without SoxN overexpression (negative for anti-V5 signal). This was carried out in late L3 larval optic lobes for SoxN-V5 and Vp16-SoxN-V5, and in adult optic lobes for EnR-SoxN-V5 overexpression experiments, because only at these stages could we clearly find T4/T5 somata with very high anti-V5 signal and neighbouring T4/T5 somata without anti-V5 signal.

Lobula plate volume was quantified as follows: for each optic lobe mounted in a posterior orientation, a *z*-stack of the entire lobula plate (labelled with anti-DN-Cadherin) was acquired with a *z*-step of 2 µm. In each optical section, the area of the manually segmented lobula plate was measured. The areas obtained from all optical sections were summed to estimate the 3D volume of the lobula plate.

The extension of lobula plate along the anteroposterior axis occupied by individual T4 and T5 axons was quantified as follows: for each lobula plate mounted in a dorsal orientation, individual axons were identified. For each axon, we measured the distance between each of its axonal boutons and the most anterior edge of the lobula plate in single optical sections. These values were normalised by the length of the lobula plate along the anteroposterior axis at the proximodistal position occupied by the axon. Finally, the normalised values of the most anterior and the most posterior axonal boutons were subtracted.

Connectin expression levels were measured as follows: for each optic lobe imaged in a dorsal orientation, we first averaged the mean grey values (anti-Connectin channel) measured in a manually defined neuropil layer across several optical sections. Next, to detect changes between experimental conditions in Connectin expression in a specific neuropil layer, for instance medulla M10, we divided Connectin levels in M10 by Connectin levels in M9, which was unchanged between conditions. For Fig. 7M-O, Connectin levels in M10 were only measured in the regions occupied by memGFP T4 dendrites.

Calculations were performed and plots were generated using Microsoft Excel Software and Python 2.7 using the NumPy and Scipy libraries. In box-and-whisker plots, the end of the whiskers represent the minimum and maximum values, and outliers were plotted as individual points. Statistical significance was assessed by calculating the *P*-value for unpaired two-tailed Student's *t*-test (*P<0.05; **P<0.01; ***P<0.001). Figures were prepared using Inkscape software.

## Supplementary Material

Supplementary information

## References

[DEV169763C1] Ammer G., Leonhardt A., Bahl A., Dickson B. J. and Borst A. (2015). Functional specialization of neural input elements to the Drosophila on motion detector. *Curr. Biol.* 25, 2247-2253. 10.1016/j.cub.2015.07.014 26234212

[DEV169763C2] Apitz H. and Salecker I. (2015). A region-specific neurogenesis mode requires migratory progenitors in the Drosophila visual system. *Nat. Neurosci.* 18, 46-55. 10.1038/nn.3896 25501037 PMC4338547

[DEV169763C3] Apitz H. and Salecker I. (2018). Spatio-temporal relays control layer identity of direction-selective neuron subtypes in Drosophila. *Nat. Commun.* 9, 2295 10.1038/s41467-018-04592-z 29895891 PMC5997761

[DEV169763C4] Bahl A., Ammer G., Schilling T. and Borst A. (2013). Object tracking in motion-blind flies. *Nat. Neurosci.* 16, 730-738. 10.1038/nn.3386 23624513

[DEV169763C5] Bahrampour S., Gunnar E., Jonsson C., Ekman H. and Thor S. (2017). Neural lineage progression controlled by a temporal proliferation program. *Dev. Cell* 43, 332-348.e334. 10.1016/j.devcel.2017.10.004 29112852

[DEV169763C6] Boergens K. M., Kapfer C., Helmstaedter M., Denk W. and Borst A. (2018). Full reconstruction of large lobula plate tangential cells in Drosophila from a 3D EM dataset. *PLoS ONE* 13, e0207828 10.1371/journal.pone.0207828 30485333 PMC6261601

[DEV169763C7] Contreras E. G., Palominos T., Glavic A., Brand A. H., Sierralta J. and Oliva C. (2018). The transcription factor SoxD controls neuronal guidance in the Drosophila visual system. *Sci. Rep.* 8, 13332 10.1038/s41598-018-31654-5 30190506 PMC6127262

[DEV169763C8] Davis F. P., Nern A., Picard S., Reiser M. B., Rubin G. M., Eddy S. R. Henry G. L. (2018). A genetic, genomic, and computational resource for exploring neural circuit function. *bioRxiv*. 10.2139/ssrn.3232155 PMC7034979 31939737

[DEV169763C9] Dietzl G., Chen D., Schnorrer F., Su K. C., Barinova Y., Fellner M., Gasser B., Kinsey K., Oppel S., Scheiblauer S. et al. (2007). A genome-wide transgenic RNAi library for conditional gene inactivation in Drosophila. *Nature* 448, 151-156. 10.1038/nature05954 17625558

[DEV169763C10] Dionne H., Hibbard K. L., Cavallaro A., Kao J. C. and Rubin G. M. (2018). Genetic reagents for making split-GAL4 lines in Drosophila. *Genetics* 209, 31-35. 10.1534/genetics.118.300682 29535151 PMC5937193

[DEV169763C11] Erclik T., Li X., Courgeon M., Bertet C., Chen Z., Baumert R., Ng J., Koo C., Arain U., Behnia R. et al. (2017). Integration of temporal and spatial patterning generates neural diversity. *Nature* 541, 365-370. 10.1038/nature20794 28077877 PMC5489111

[DEV169763C12] Ferrero E., Fischer B. and Russell S. (2014). SoxNeuro orchestrates central nervous system specification and differentiation in Drosophila and is only partially redundant with Dichaete. *Genome Biol.* 15, R74 10.1186/gb-2014-15-5-r74 24886562 PMC4072944

[DEV169763C13] Fischbach K.-F. and Dittrich A. P. M. (1989). The optic lobe of Drosophila melanogaster. I. A Golgi analysis of wild-type structure. *Cell Tissue Res.* 258, 441-475. 10.1007/BF00218858

[DEV169763C14] Gao S., Takemura S.-Y., Ting C.-Y., Huang S., Lu Z., Luan H., Rister J., Thum A. S., Yang M., Hong S. T. et al. (2008). The neural substrate of spectral preference in Drosophila. *Neuron* 60, 328-342. 10.1016/j.neuron.2008.08.010 18957224 PMC2665173

[DEV169763C15] Girard F., Joly W., Savare J., Bonneaud N., Ferraz C. and Maschat F. (2006). Chromatin immunoprecipitation reveals a novel role for the Drosophila SoxNeuro transcription factor in axonal patterning. *Dev. Biol.* 299, 530-542. 10.1016/j.ydbio.2006.08.014 16979619

[DEV169763C16] Haag J., Arenz A., Serbe E., Gabbiani F. and Borst A. (2016). Complementary mechanisms create direction selectivity in the fly. *eLife* 5, e17421 10.7554/eLife.17421 27502554 PMC4978522

[DEV169763C17] Haag J., Mishra A. and Borst A. (2017). A common directional tuning mechanism of Drosophila motion-sensing neurons in the ON and in the OFF pathway. *eLife* 6, e29044 10.7554/eLife.29044 28829040 PMC5582866

[DEV169763C18] Hasegawa E., Kaido M., Takayama R. and Sato M. (2013). Brain-specific-homeobox is required for the specification of neuronal types in the Drosophila optic lobe. *Dev. Biol.* 377, 90-99. 10.1016/j.ydbio.2013.02.012 23454478

[DEV169763C19] Hasegawa E., Kitada Y., Kaido M., Takayama R., Awasaki T., Tabata T. and Sato M. (2011). Concentric zones, cell migration and neuronal circuits in the Drosophila visual center. *Development* 138, 983-993. 10.1242/dev.058370 21303851

[DEV169763C20] Hobert O. (2011). Regulation of terminal differentiation programs in the nervous system. *Annu. Rev. Cell Dev. Biol.* 27, 681-696. 10.1146/annurev-cellbio-092910-154226 21985672

[DEV169763C21] Hobert O. (2016). Terminal selectors of neuronal identity. *Curr. Top. Dev. Biol.* 116, 455-475. 10.1016/bs.ctdb.2015.12.007 26970634

[DEV169763C22] Hofbauer A. and Campos-Ortega J. A. (1990). Proliferation pattern and early differentiation of the optic lobes in Drosophila melanogaster. *Roux's Arch. Dev. Biol.* 198, 264-274. 10.1007/BF00377393 28305665

[DEV169763C23] Jarman A. P., Grell E. H., Ackerman L., Jan L. Y. and Jan Y. N. (1994). Atonal is the proneural gene for Drosophila photoreceptors. *Nature* 369, 398-400. 10.1038/369398a0 8196767

[DEV169763C24] Joesch M., Plett J., Borst A. and Reiff D. F. (2008). Response properties of motion-sensitive visual interneurons in the lobula plate of Drosophila melanogaster. *Curr. Biol.* 18, 368-374. 10.1016/j.cub.2008.02.022 18328703

[DEV169763C25] Joesch M., Schnell B., Raghu S. V., Reiff D. F. and Borst A. (2010). ON and OFF pathways in Drosophila motion vision. *Nature* 468, 300-304. 10.1038/nature09545 21068841

[DEV169763C26] Kaya-Copur A. and Schnorrer F. (2016). A guide to genome-wide in vivo RNAi applications in Drosophila. *Methods Mol. Biol.* 1478, 117-143. 10.1007/978-1-4939-6371-3_6 27730578

[DEV169763C27] Kitamoto T. (2001). Conditional modification of behavior in Drosophila by targeted expression of a temperature-sensitive shibire allele in defined neurons. *J. Neurobiol.* 47, 81-92. 10.1002/neu.1018 11291099

[DEV169763C28] Konstantinides N., Kapuralin K., Fadil C., Barboza L., Satija R. and Desplan C. (2018). Phenotypic convergence: distinct transcription factors regulate common terminal features. *Cell* 174, 622-635.e13. 10.1016/j.cell.2018.05.021 29909983 PMC6082168

[DEV169763C29] Kwan K. Y., Lam M. M., Krsnik Z., Kawasawa Y. I., Lefebvre V. and Sestan N. (2008). SOX5 postmitotically regulates migration, postmigratory differentiation, and projections of subplate and deep-layer neocortical neurons. *Proc. Natl. Acad. Sci. USA* 105, 16021-16026. 10.1073/pnas.0806791105 18840685 PMC2572944

[DEV169763C30] Lai T., Jabaudon D., Molyneaux B. J., Azim E., Arlotta P., Menezes J. R. and Macklis J. D. (2008). SOX5 controls the sequential generation of distinct corticofugal neuron subtypes. *Neuron* 57, 232-247. 10.1016/j.neuron.2007.12.023 18215621

[DEV169763C31] Li X., Erclik T., Bertet C., Chen Z., Voutev R., Venkatesh S., Morante J., Celik A. and Desplan C. (2013). Temporal patterning of Drosophila medulla neuroblasts controls neural fates. *Nature* 498, 456-462. 10.1038/nature12319 23783517 PMC3701960

[DEV169763C32] Li A., Hooli B., Mullin K., Tate R. E., Bubnys A., Kirchner R., Chapman B., Hofmann O., Hide W. and Tanzi R. E. (2017). Silencing of the Drosophila ortholog of SOX5 leads to abnormal neuronal development and behavioral impairment. *Hum. Mol. Genet.* 26, 1472-1482. 10.1093/hmg/ddx051 28186563 PMC6075463

[DEV169763C33] Lodato S. and Arlotta P. (2015). Generating neuronal diversity in the mammalian cerebral cortex. *Annu. Rev. Cell Dev. Biol.* 31, 699-720. 10.1146/annurev-cellbio-100814-125353 26359774 PMC4778709

[DEV169763C34] Maisak M. S., Haag J., Ammer G., Serbe E., Meier M., Leonhardt A., Schilling T., Bahl A., Rubin G. M., Nern A. et al. (2013). A directional tuning map of Drosophila elementary motion detectors. *Nature* 500, 212-216. 10.1038/nature12320 23925246

[DEV169763C35] Mauss A. S., Meier M., Serbe E. and Borst A. (2014). Optogenetic and pharmacologic dissection of feedforward inhibition in Drosophila motion vision. *J. Neurosci.* 34, 2254-2263. 10.1523/JNEUROSCI.3938-13.2014 24501364 PMC6608528

[DEV169763C36] Mauss A. S., Pankova K., Arenz A., Nern A., Rubin G. M. and Borst A. (2015). Neural circuit to integrate opposing motions in the visual field. *Cell* 162, 351-362. 10.1016/j.cell.2015.06.035 26186189

[DEV169763C37] Mora N., Oliva C., Fiers M., Ejsmont R., Soldano A., Zhang T. T., Yan J., Claeys A., De Geest N. and Hassan B. A. (2018). A temporal transcriptional switch governs stem cell division, neuronal numbers, and maintenance of differentiation. *Dev. Cell* 45, 53-66.e55. 10.1016/j.devcel.2018.02.023 29576424

[DEV169763C38] Nassif C., Noveen A. and Hartenstein V. (2003). Early development of the Drosophila brain: III. The pattern of neuropile founder tracts during the larval period. *J. Comp. Neurol.* 455, 417-434. 10.1002/cne.10482 12508317

[DEV169763C39] Neriec N. and Desplan C. (2014). Different ways to make neurons: parallel evolution in the SoxB family. *Genome Biol.* 15, 116 10.1186/gb4177 25001546 PMC4072935

[DEV169763C40] Nern A., Pfeiffer B. D., Svoboda K. and Rubin G. M. (2011). Multiple new site-specific recombinases for use in manipulating animal genomes. *Proc. Natl. Acad. Sci. USA* 108, 14198-14203. 10.1073/pnas.1111704108 21831835 PMC3161616

[DEV169763C41] Nern A., Pfeiffer B. D. and Rubin G. M. (2015). Optimized tools for multicolor stochastic labeling reveal diverse stereotyped cell arrangements in the fly visual system. *Proc. Natl. Acad. Sci. USA* 112, E2967-E2976. 10.1073/pnas.1506763112 25964354 PMC4460454

[DEV169763C42] Ngo K. T., Andrade I. and Hartenstein V. (2017). Spatio-temporal pattern of neuronal differentiation in the Drosophila visual system: a user's guide to the dynamic morphology of the developing optic lobe. *Dev. Biol.* 428, 1-24. 10.1016/j.ydbio.2017.05.008 28533086 PMC5825191

[DEV169763C43] Nose A., Umeda T. and Takeichi M. (1997). Neuromuscular target recognition by a homophilic interaction of connectin cell adhesion molecules in Drosophila. *Development* 124, 1433-1441. 9108360 10.1242/dev.124.8.1433

[DEV169763C44] Oliva C., Choi C. M., Nicolai L. J., Mora N., De Geest N. and Hassan B. A. (2014). Proper connectivity of Drosophila motion detector neurons requires Atonal function in progenitor cells. *Neural Dev.* 9, 4 10.1186/1749-8104-9-4 24571981 PMC3941608

[DEV169763C45] Overton P. M., Meadows L. A., Urban J. and Russell S. (2002). Evidence for differential and redundant function of the Sox genes Dichaete and SoxN during CNS development in Drosophila. *Development* 129, 4219-4228. 12183374 10.1242/dev.129.18.4219

[DEV169763C46] Pankova K. and Borst A. (2016). RNA-Seq transcriptome analysis of direction-selective T4/T5 neurons in Drosophila. *PLoS ONE* 11, e0163986 10.1371/journal.pone.0163986 27684367 PMC5042512

[DEV169763C47] Peng J., Santiago I. J., Ahn C., Gur B., Tsui C. K., Su Z., Xu C., Karakhanyan A., Silies M. and Pecot M. Y. (2018). Drosophila Fezf coordinates laminar-specific connectivity through cell-intrinsic and cell-extrinsic mechanisms. *eLife* 7, e33962 10.7554/eLife.33962 29513217 PMC5854465

[DEV169763C48] Perkins L. A., Holderbaum L., Tao R., Hu Y., Sopko R., McCall K., Yang-Zhou D., Flockhart I., Binari R., Shim H. S. et al. (2015). The transgenic RNAi project at harvard medical school: resources and validation. *Genetics* 201, 843-852. 10.1534/genetics.115.180208 26320097 PMC4649654

[DEV169763C49] Pfeiffer B. D., Ngo T. T., Hibbard K. L., Murphy C., Jenett A., Truman J. W. and Rubin G. M. (2010). Refinement of tools for targeted gene expression in Drosophila. *Genetics* 186, 735-755. 10.1534/genetics.110.119917 20697123 PMC2942869

[DEV169763C50] Phochanukul N. and Russell S. (2010). No backbone but lots of Sox: invertebrate Sox genes. *Int. J. Biochem. Cell Biol.* 42, 453-464. 10.1016/j.biocel.2009.06.013 19589395

[DEV169763C51] Pinto-Teixeira F., Koo C., Rossi A. M., Neriec N., Bertet C., Li X., Del-Valle-Rodriguez A. and Desplan C. (2018). Development of concurrent retinotopic maps in the fly motion detection circuit. *Cell* 173, 485-498.e411. 10.1016/j.cell.2018.02.053 29576455 PMC5889347

[DEV169763C52] Raghavan S. and White R. A. (1997). Connectin mediates adhesion in Drosophila. *Neuron* 18, 873-880. 10.1016/S0896-6273(00)80327-X 9208855

[DEV169763C53] Savare J., Bonneaud N. and Girard F. (2005). SUMO represses transcriptional activity of the Drosophila SoxNeuro and human Sox3 central nervous system-specific transcription factors. *Mol. Biol. Cell* 16, 2660-2669. 10.1091/mbc.e04-12-1062 15788563 PMC1142414

[DEV169763C54] Schilling T. and Borst A. (2015). Local motion detectors are required for the computation of expansion flow-fields. *Biol. Open* 4, 1105-1108. 10.1242/bio.012690 26231626 PMC4582123

[DEV169763C55] Schindelin J., Arganda-Carreras I., Frise E., Kaynig V., Longair M., Pietzsch T., Preibisch S., Rueden C., Saalfeld S., Schmid B. et al. (2012). Fiji: an open-source platform for biological-image analysis. *Nat. Methods* 9, 676-682. 10.1038/nmeth.2019 22743772 PMC3855844

[DEV169763C56] Schnell B., Joesch M., Forstner F., Raghu S. V., Otsuna H., Ito K., Borst A. and Reiff D. F. (2010). Processing of horizontal optic flow in three visual interneurons of the Drosophila brain. *J. Neurophysiol.* 103, 1646-1657. 10.1152/jn.00950.2009 20089816

[DEV169763C57] Scott E. K., Raabe T. and Luo L. (2002). Structure of the vertical and horizontal system neurons of the lobula plate in Drosophila. *J. Comp. Neurol.* 454, 470-481. 10.1002/cne.10467 12455010

[DEV169763C58] She Z. Y. and Yang W. X. (2015). SOX family transcription factors involved in diverse cellular events during development. *Eur. J. Cell Biol.* 94, 547-563. 10.1016/j.ejcb.2015.08.002 26340821

[DEV169763C59] Shinomiya K., Karuppudurai T., Lin T. Y., Lu Z., Lee C. H. and Meinertzhagen I. A. (2014). Candidate neural substrates for off-edge motion detection in Drosophila. *Curr. Biol.* 24, 1062-1070. 10.1016/j.cub.2014.03.051 24768048 PMC4031294

[DEV169763C60] Shinomiya K., Takemura S. Y., Rivlin P. K., Plaza S. M., Scheffer L. K. and Meinertzhagen I. A. (2015). A common evolutionary origin for the ON- and OFF-edge motion detection pathways of the Drosophila visual system. *Front. Neural Circuits* 9, 33 10.3389/fncir.2015.00033 26217193 PMC4496578

[DEV169763C61] Strother J. A., Nern A. and Reiser M. B. (2014). Direct observation of ON and OFF pathways in the Drosophila visual system. *Curr. Biol.* 24, 976-983. 10.1016/j.cub.2014.03.017 24704075

[DEV169763C62] Suzuki T., Kaido M., Takayama R. and Sato M. (2013). A temporal mechanism that produces neuronal diversity in the Drosophila visual center. *Dev. Biol.* 380, 12-24. 10.1016/j.ydbio.2013.05.002 23665475

[DEV169763C63] Suzuki T., Hasegawa E., Nakai Y., Kaido M., Takayama R. and Sato M. (2016). Formation of neuronal circuits by interactions between neuronal populations derived from different origins in the Drosophila visual center. *Cell Reports* 15, 499-509. 10.1016/j.celrep.2016.03.056 27068458

[DEV169763C64] Takemura S. Y., Nern A., Chklovskii D. B., Scheffer L. K., Rubin G. M. and Meinertzhagen I. A. (2017). The comprehensive connectome of a neural substrate for ‘ON’ motion detection in Drosophila. *eLife* 6, e24394 10.7554/eLife.24394 28432786 PMC5435463

[DEV169763C65] Tirian L. and Dickson B. (2017). The VT GAL4, LexA, and split-GAL4 driver line collections for targeted expression in the Drosophila nervous system. *bioRxiv*. 10.1101/198648

[DEV169763C66] Tuthill J. C., Nern A., Holtz S. L., Rubin G. M. and Reiser M. B. (2013). Contributions of the 12 neuron classes in the fly lamina to motion vision. *Neuron* 79, 128-140. 10.1016/j.neuron.2013.05.024 23849200 PMC3806040

