# Supplementary material for: Transcriptional control of morphological properties of direction-selective T4/T5 neurons in Drosophila
Source: Development. 2019 Jan 29;146(2):dev169763. doi: 10.1242/dev.169763 (PMC6361130; doi:10.1242/dev.169763)
Supplement: Supplementary information [file develop-146-169763-s1.pdf]

Development: doi:10.1242/dev.169763: Supplementary information

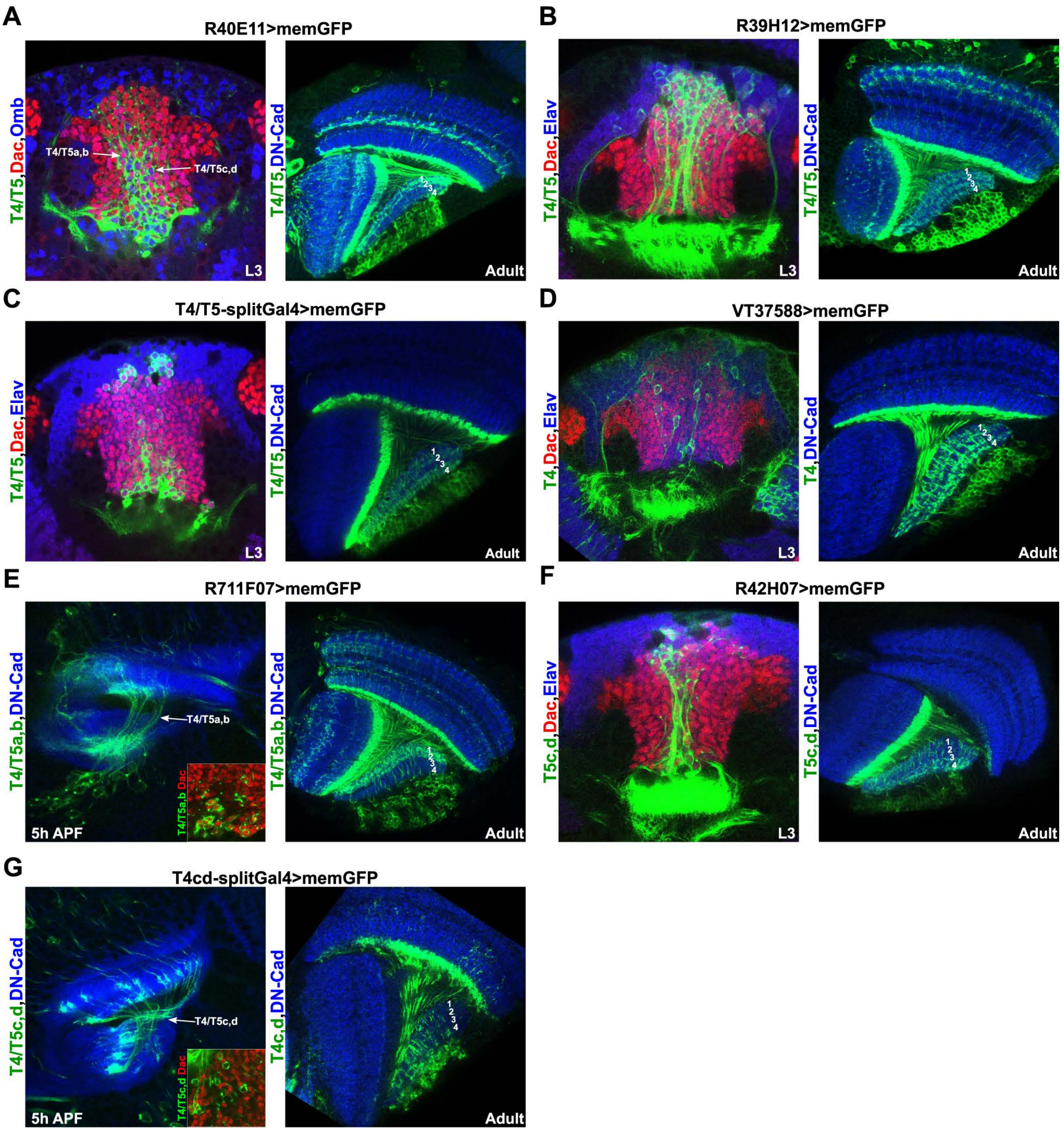

Figure S1. Characterization of *enhancer-Gal4* driver lines used for *UAS-RNAi* transgene expression in T4/T5 neurons.

(A-G) Expression patterns of *R40E11-Gal4*, *R39H12-Gal4*, *T4/T5-splitGal4, VT37588-Gal4*, *R11F07-Gal4*, *R42H07-Gal4* and *T4$_{c,d}$-splitGal4* driver lines in late L3 larval or early pupal optic lobes (5 h APF), and in adult optic lobes. Arrows in A mark Dac$^+$/Omb$^-$ and Dac$^-$/Omb$^+$ somata, which correspond to T4/T5$_{a,b}$ and T4/T5$_{c,d}$ neurons, respectively. Inset in E shows that most T4/T5 somata labelled by the *R11F07-Gal4* at 5 h APF are Dac$^+$. Inset in G shows that most T4/T5 somata labelled by the *T4$_{c,d}$-splitGal4* at 5 h APF are Dac$^-$. Neuropils were labelled with anti-DN-Cad at pupal and adult stages.

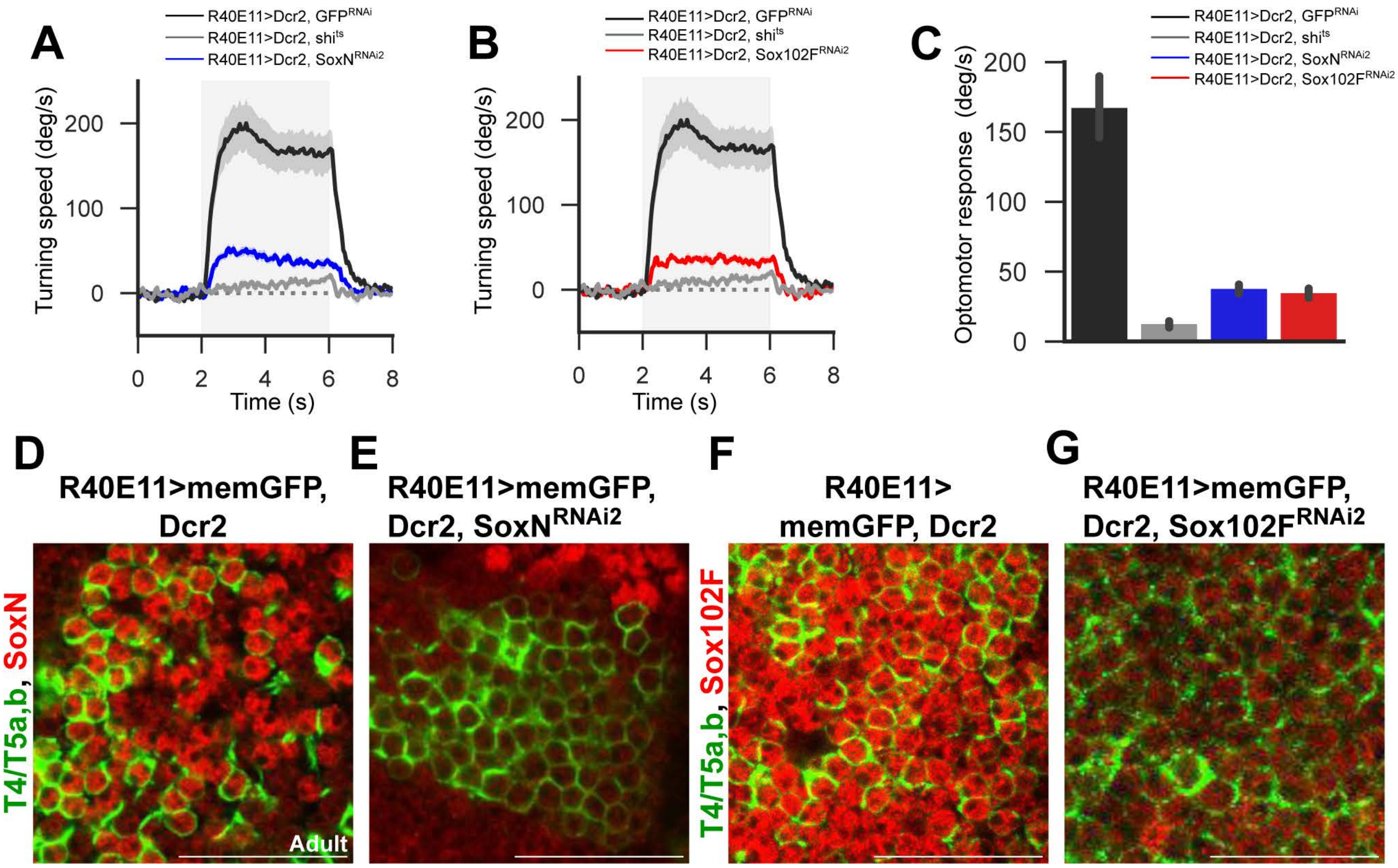

Figure S2. Use of additional *UAS-RNAi* transgenes supports that knockdown of *SoxN* or *Sox102F* in T4/T5 neurons impairs the optomotor response.

(A,B) Average turning speeds in response to rotation of a grating pattern (grey shaded areas) of flies expressing *GFP-RNAi* (negative control), *shi*^{ts} (positive control, T4/T5 block), *SoxN-RNAi2* or *Sox102F-RNAi2* in T4/T5 neurons (n = 10 flies per group).

(C) Average optomotor responses of flies expressing *GFP-RNAi*, *shi*^{ts}, *SoxN-RNAi2* or *Sox102F-RNAi2* in T4/T5 neurons (n = 10 flies per group).

(D-G) SoxN and Sox102F expression in adult optic lobes with wild-type T4/T5 neurons, and with T4/T5 neurons expressing *SoxN-RNAi2* or *Sox102F-RNAi2.*

Scale bars = 20 µm.

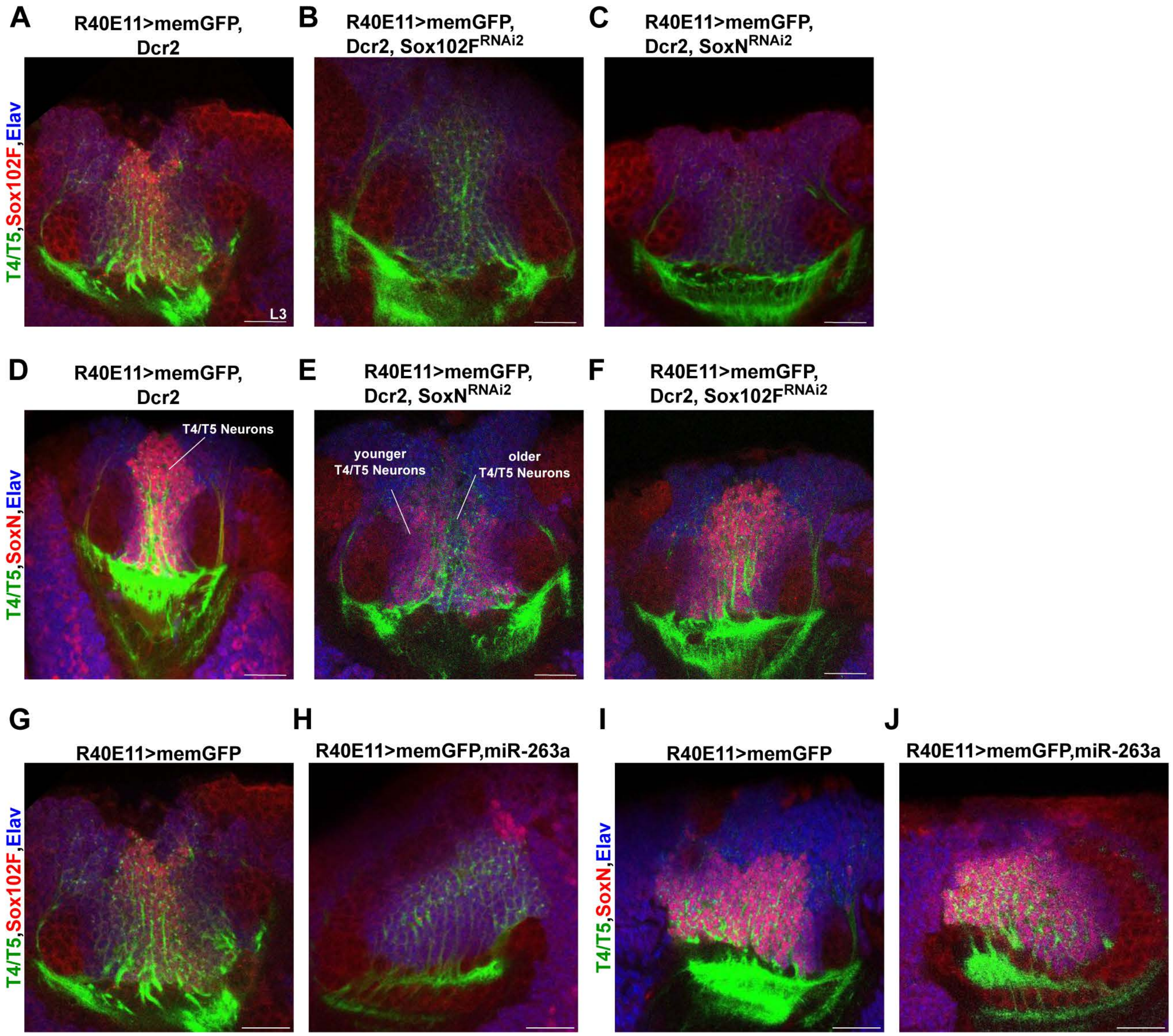

Figure S3. Use of additional *UAS-RNAi* transgenes and microRNA supports that *SoxN* is required for Sox102F expression while *Sox102F* is dispensable for SoxN expression in T4/T5 neurons.

(A-C) Sox102F expression in late L3 larval optic lobes with wild-type T4/T5 neurons, and with T4/T5 neurons expressing *Sox102F-RNAi2* or *SoxN-RNAi2.*

(D-F) SoxN expression in late L3 larval optic lobes with wild-type T4/T5 neurons and with T4/T5 neurons expressing *SoxN-RNAi2* or *Sox102F-RNAi2.*

(G-J) Sox102F and SoxN expression in in late L3 larval optic lobes with wild-type T4/T5 neurons, and with T4/T5 neurons expressing *miR-263a.*

Scale bars = 20 µm.

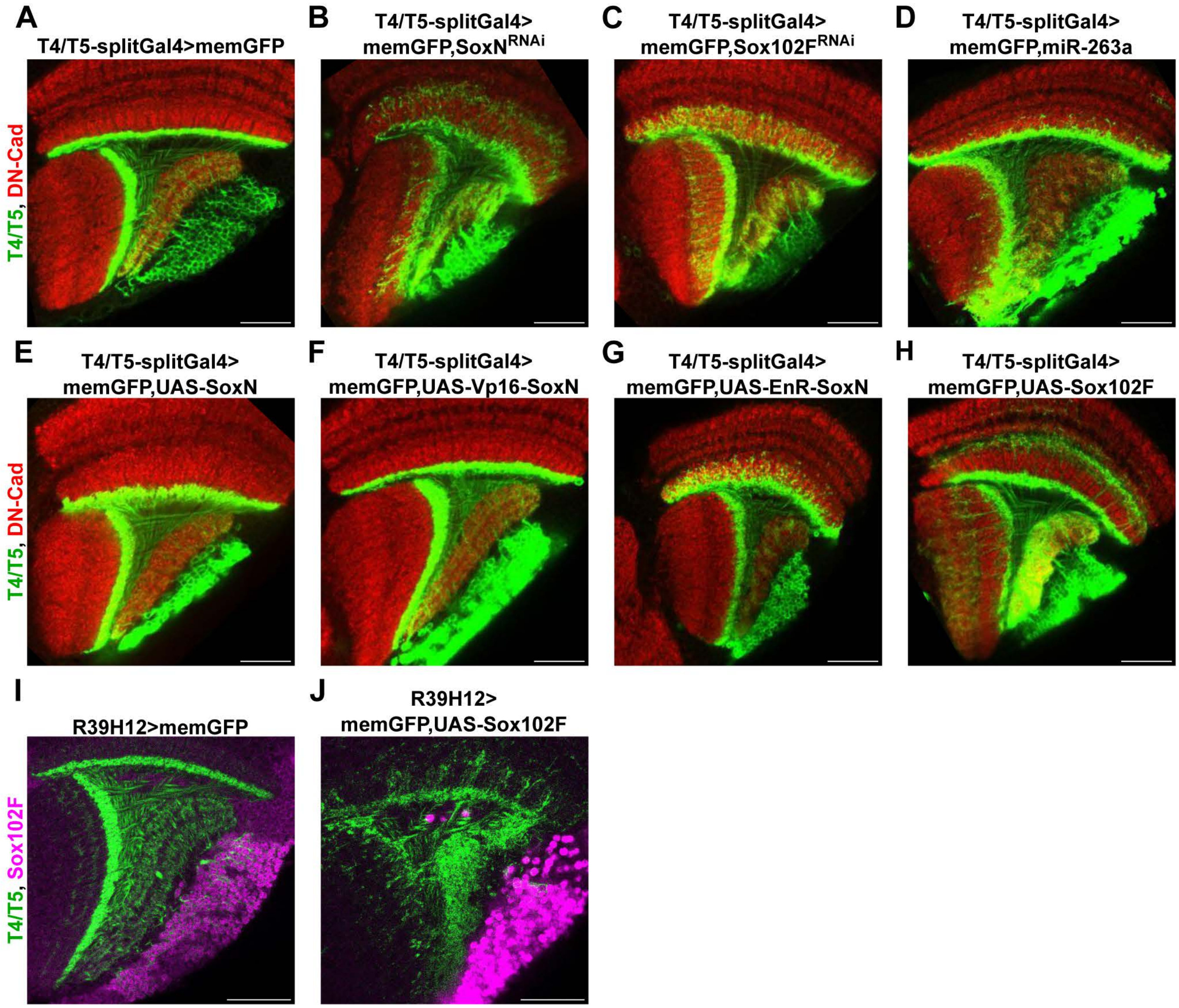

Figure S4. T4/T5 neuron morphology upon *SoxN* or *Sox102F* silencing, and upon SoxN or Sox102F overexpression with the *T4/T5-splitGal4* driver line.

(A-D) Adult optic lobes with wild-type T4/T5 neurons, and with T4/T5 neurons expressing *SoxN-RNAi, Sox102F-RNAi* or *miR-263a* by means of the *T4/T5-splitGal4* line.

(E-G) Adult optic lobes with T4/T5 neurons overexpressing three different versions of SoxN by using the *T4/T5-splitGal4* line: a wild-type version (SoxN), an obligatory activator version (Vp16-SoxN), or an obligatory repressor version (EnR-SoxN).

(H) Adult optic lobe with T4/T5 neurons overexpressing Sox102F by means of the *T4/T5-splitGal4* line.

(I-J) Sox102F expression in adult optic lobes with wild-type T4/T5 neurons, and with T4/T5 neurons overexpressing Sox102F by means of the *R39H12-Gal4* line.

Scale bars = 20 µm.

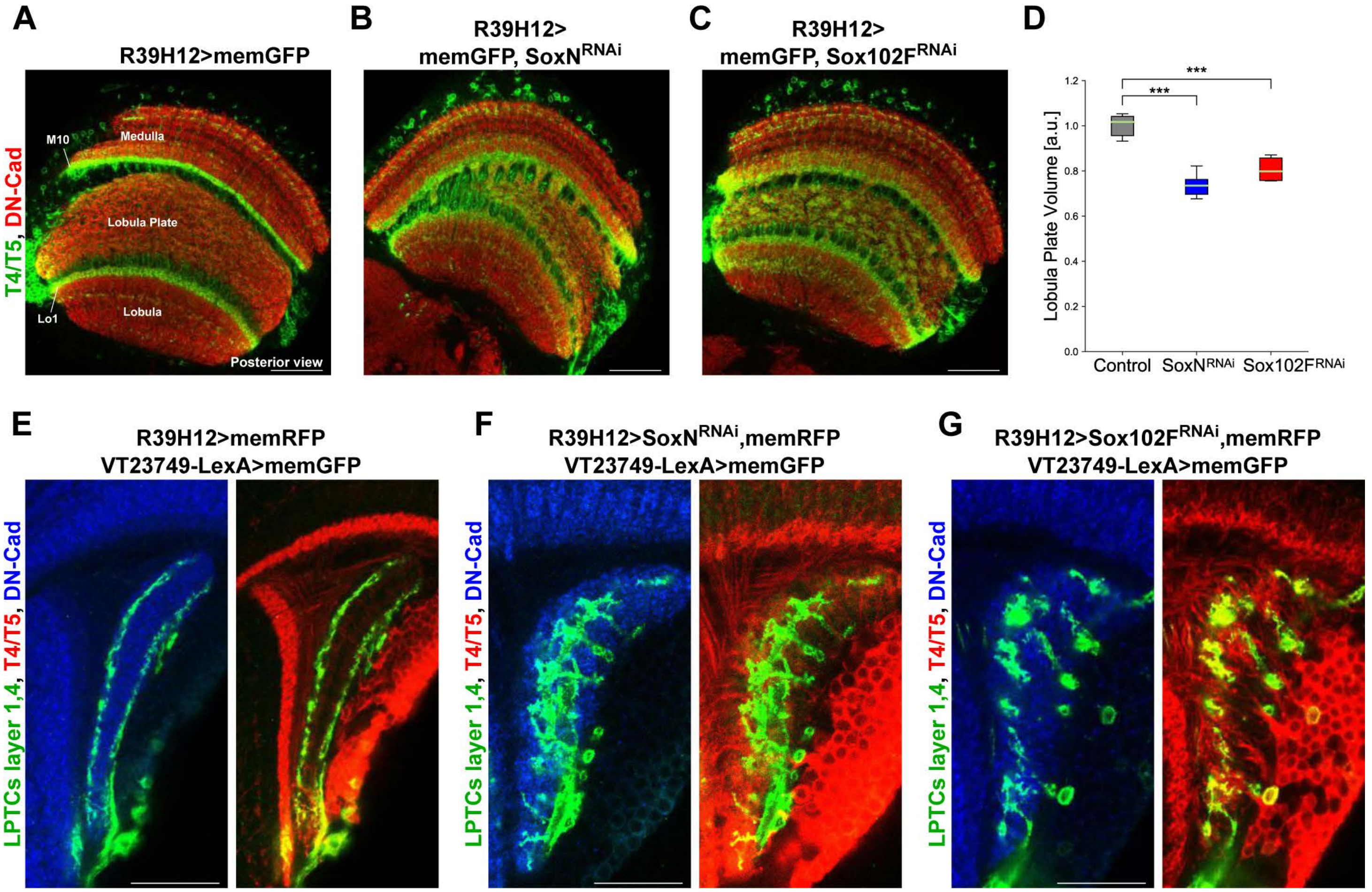

Figure S5. *SoxN* and *Sox102F* control dendritic development non-autonomously in LPTCs.

(A-C) Posterior views of adult optic lobes with wild-type T4/T5 neurons, and with T4/T5 neurons expressing *SoxN-RNAi* or *Sox102F-RNAi*.

(D) Average lobula plate volumes in adult flies with wild-type T4/T5 neurons (control), and with T4/T5 neurons expressing *SoxN-RNAi* or *Sox102F-RNAi* (n = 5 optic lobes per group). ****P*<0.001.

(E-G) Dorsal view of lobula plates showing the dendrites of LPTCs in the presence of wild-type T4/T5 axons, and in the presence of T4/T5 axons expressing *SoxN-RNAi* or *Sox102F-RNAi*.

Scale bars: A-C = 40 μm, E-G = 20 μm.

Development • Supplementary information

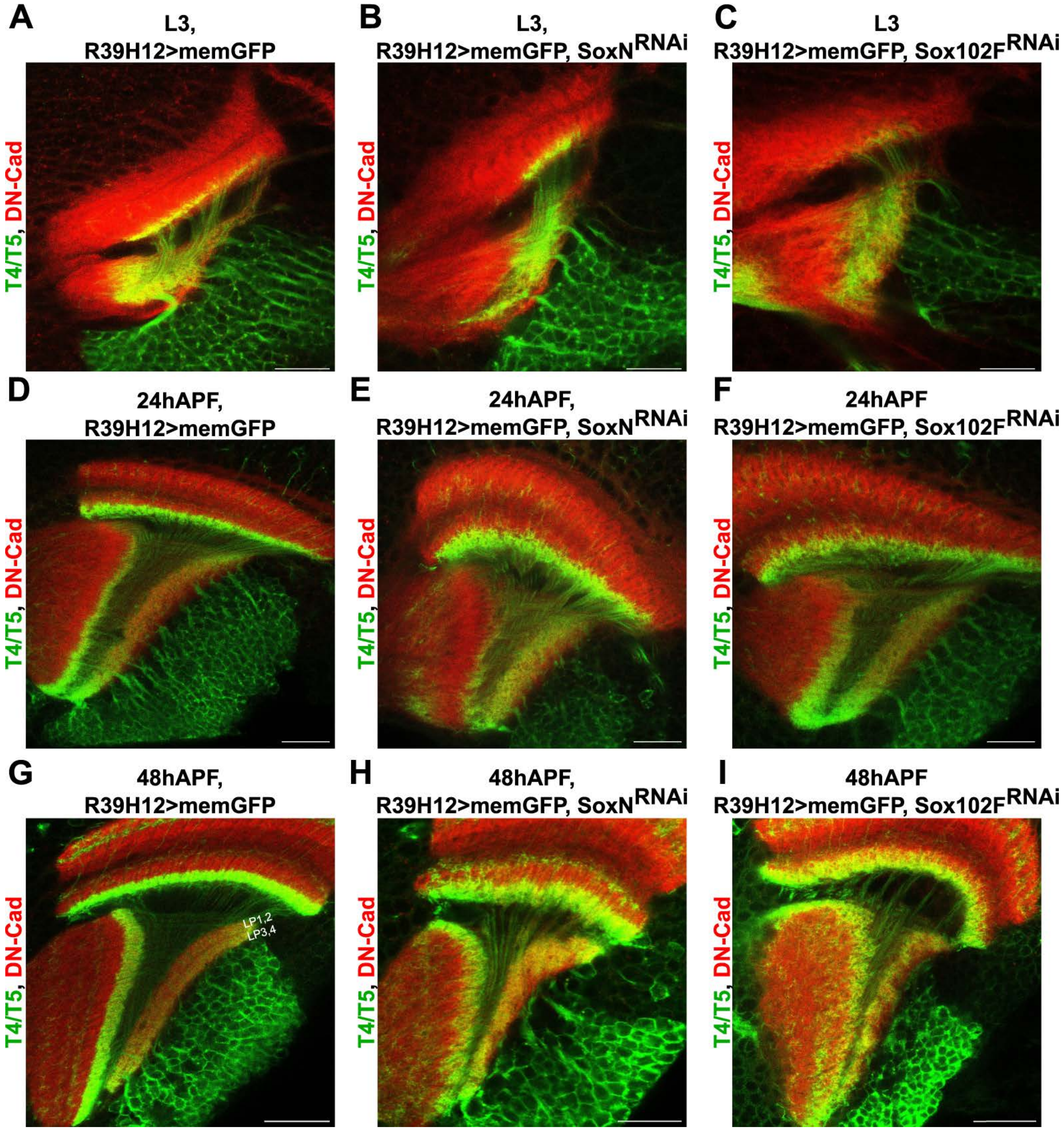

Figure S6. T4/T5 neuron morphology during development upon silencing *SoxN* and *Sox102F* with the *R39H12-Gal4* line.

(A-I) Dorsal views of optic lobes at late L3 larval stage, and at pupal stages 24 hours APF and 48 hours APF with wild-type T4/T5 neurons, and with T4/T5 neurons expressing *SoxN-RNAi* or *Sox102F-RNAi*.

Scale bars: A-C = 10 μm, D-I = 20 μm.

Development • Supplementary information

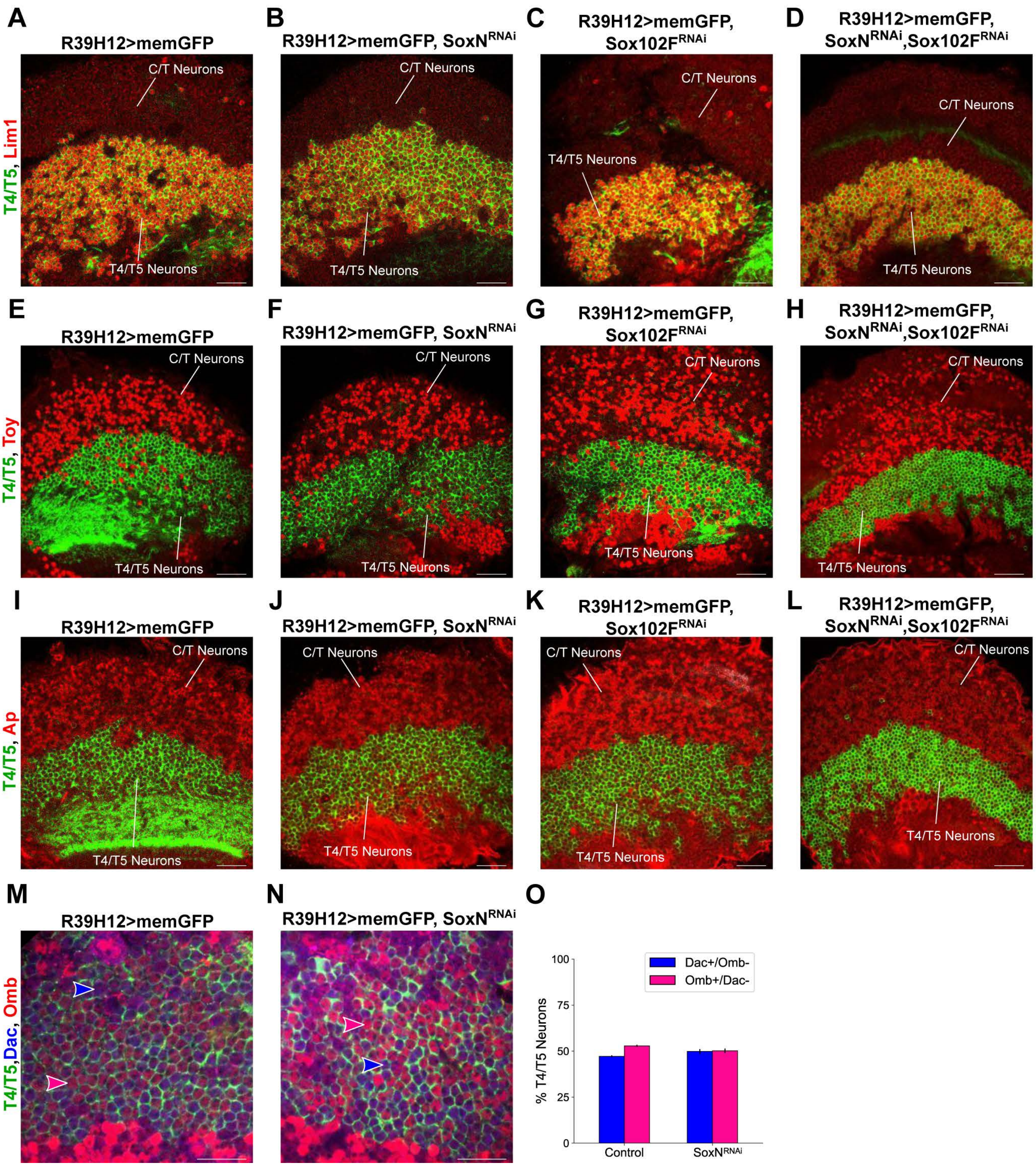

Development • Supplementary information

Figure S7. Lim1, Toy, Ap, Dac and Omb expression in optic lobes with wild-type T4/T5 neurons, and with T4/T5 neurons with *SoxN* and *Sox102F* silenced.

(A-L) Lim1, Toy and Ap expression in the region occupied by T4/T5 and C/T somata at adult stage. Wild-type T4/T5 somata, and somata from T4/T5 neurons expressing *SoxN-RNAi*, *Sox102F-RNAi, or SoxN-RNAi and Sox102F-RNAi* together were labelled with memGFP.

(M-O) Dac and Omb expression in adult, wild-type T4/T5 somata, and in adult, T4/T5 somata after knockdown of *SoxN*. Blue and magenta arrowheads indicate Dac^{+}/Omb^{-} and Dac^{-}/Omb^{+} somata, respectively. The percentages of Dac^{+}/Omb^{-} ($T4/T5_{a,b}$) and Dac^{-}/Omb^{+} ($T4/T5_{c,d}$) neurons in each condition are shown in O (n = 5 optic lobes per group, 150-200 somata examined per optic lobe).

Scale bars = 20 μm.

Development • Supplementary information

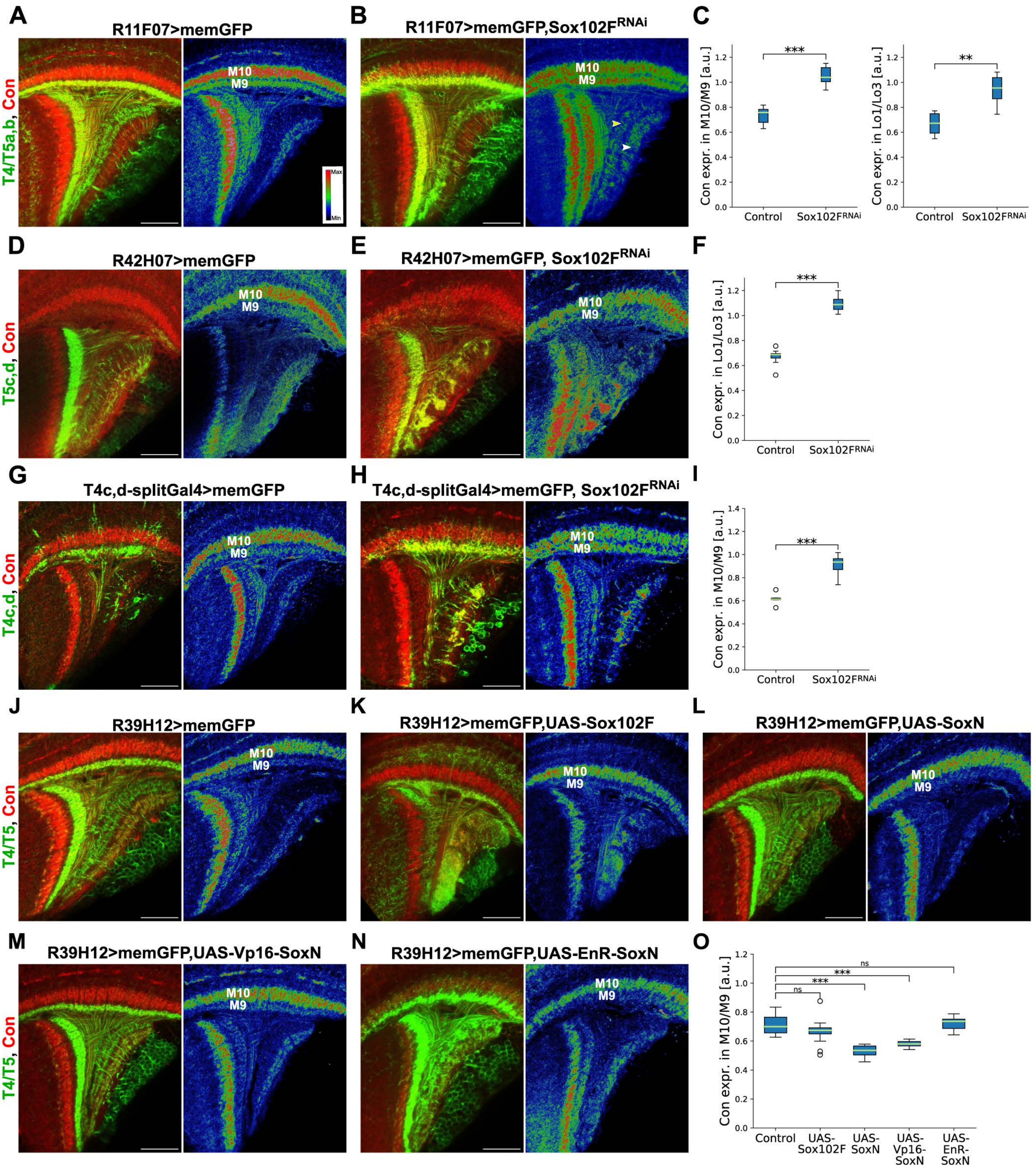

Development • Supplementary information

Figure S8. Connectin levels upon overexpression of SoxN or Sox102F in T4/T5 neurons, and upon silencing of *SoxN* or *Sox102F* in distinct T4/T5 neuron subtypes.

(A-C) Connectin expression in adult optic lobes with wild-type T4/T5$_{a,b}$ neurons, and with T4/T5$_{a,b}$ neurons expressing *Sox102F-RNAi*. Axons from T4/T5$_{a,b}$ neurons with *Sox102F* knockdown show similar Connectin levels (yellow arrowhead in B) than axons from wild-type T4/T5$_{c,d}$ neurons (white arrowhead in B). Ratios of Connectin signal in medulla layer M10 to Connectin signal in medulla layer M9, and ratios of Connectin signal in lobula layer Lo1 to Connectin signal in lobula layer Lo3 are shown in C (n = 8-13 optic lobes per group). The right panels show Connectin signals colour coded for intensity.

(D-F) Connectin expression in adult optic lobes with wild-type T5$_{c,d}$ neurons, and with T5$_{c,d}$ neurons expressing *Sox102F-RNAi*. Ratios of Connectin signal in lobula layer Lo1 to Connectin signal in lobula layer Lo3 are shown in F (n = 8-11 optic lobes per group).

(G-I) Connectin expression in adult optic lobes with wild-type T4$_{c,d}$ neurons, and with T4$_{c,d}$ neurons expressing *Sox102F-RNAi*. Ratios of Connectin signal in medulla layer M10 to Connectin signal in medulla layer M9 are shown in I (n = 6-8 optic lobes per group).

(J-O) Connectin expression in adult optic lobes with wild-type T4/T5 neurons, and with T4/T5 neurons overexpressing Sox102F, SoxN (wild-type version), Vp16-SoxN (obligatory activator version) or EnR-SoxN (obligatory repressor version). Ratios of Connectin signal in medulla layer M10 to Connectin signal in medulla layer M9 are shown in O (n = 7-14 optic lobes per group). ns, not significant (P>0.05); **P<0.01; ***P<0.001.

Scale bars = 20 µm.

Development • Supplementary information
